# Oestrogen and growth factor cross-talk and endocrine insensitivity and acquired resistance in breast cancer

**DOI:** 10.1054/bjoc.1999.0954

**Published:** 2000-01-18

**Authors:** RI Nicholson, J M W Gee

**Affiliations:** Tenovus Cancer Research Centre, University of Wales College of Medicine, Cardiff, CF14 4XX, UK


*British Journal of Cancer* (2000) **82**(3), 501–513
© 2000 Cancer Research Campaign
DOI: 10.1054/ bjoc.1999.0954, available online at http://www.idealibrary.com on IDEAL®

Review

### Oestrogen and growth factor cross-talk and endocrine insensitivity and acquired resistance in breast cancer

**RI Nicholson and JMW Gee**

Tenovus Cancer Research Centre, University of Wales College of Medicine, Cardiff CF14 4XX, UK

It is a sobering thought that as we approach the 21st century, breast cancer remains one of the most prevalent of all carcinomas, with one in eight women in Western societies being expected to develop the disease at some point in her life. Research examining those factors affecting the development of breast cancer has identified that steroid hormones are of pivotal importance in directing the growth of these tumours. This knowledge has been exploited clinically, with endocrine treatments which seek to perturb the steroid hormone environment of the tumour cells often promoting extensive remissions in established tumours and furthermore providing significant survival benefits for patients (Nicholson et al, 1992). Unfortunately, the beneficial actions of existing endocrine measures are, in part, counteracted by the capacity of the tumour cells to eventually circumvent the need for steroid hormones, allowing them to continue to grow and progress despite such therapy (Gee et al, 1996; Nicholson et al, 1996). Thus, at presentation of breast cancer, current endocrine therapies are not effective in all patients (de novo endocrine resistance), while initially-responsive tumours will sooner or later progress despite such treatments (acquired resistance), inevitably resulting in patient relapse and, ultimately, death. Identification of the factors and pathways responsible for the development of these resistant conditions is therefore an important diagnostic and therapeutic goal in cancer research.

One proposed model for such loss of steroid hormone sensitivity in breast cancer in both the de novo and acquired setting suggests that aberrations advantageous to tumour cell growth occur specifically within important growth factor signalling pathways, allowing mitogenesis to proceed highly efficiently despite the challenge of endocrine therapy. A new paradigm is thus emerging where knowledge of the tumour expression of growth factor signalling elements may be prognostically relevant in identifying endocrine responsiveness, and where appropriate anti-growth factor signalling therapeutic regimens, in combination with antihormonal measures, would be expected to be beneficial to breast cancer patients (Nicholson et al, 1999*a*).

In this light, the present review seeks to outline the elaborate molecular biology of oestrogen and growth factor signalling pathway interactions which are likely to play a central role in hormone sensitive breast tumour growth. It subsequently examines how changes often occurrent in the breast cancer phenotype might severely perturb the balance of such signalling, thus providing a possible explanatory hypothesis for the tumour growth associated with the phenomena of de novo and acquired endocrine resistance. A discussion of how such data might be therapeutically-exploitable in breast cancer has been published elsewhere (Nicholson et al, 1999*a*, 1999*b*).

*Received 15 March 1999*
*Revised 11 June 1999*
*Accepted 8 August 1999*

*Correspondence to:* RI Nicholson

### 'Cross-talk' between steroid hormone and growth factor signalling pathways influences the growth of endocrine responsive disease

Many studies have now identified that breast tumours which exhibit an effective endocrine response (i.e. complete and partial response) are often histologically low grade, well-differentiated and notably oestrogen receptor (ER)-positive with a minimal level of proliferation at presentation (Williams et al, 1986; Bouzubar et al, 1989; Robertson et al, 1989; Nicholson et al, 1991, 1993; Locker et al, 1992; Cheung et al, 1997). The 40–50% of breast cancer patients bearing such tumours frequently enjoy a long duration of response and survival time (Nicholson et al, 1984). In such tumours, it is likely that ER signalling is central to mitogenesis, with steroid hormone occupancy of the receptor efficiently driving cell growth and survival together with expression of target genes bearing either oestrogen response elements (ERE) (Nicholson et al, 1999*a*, 1999*b*; Seery et al, in press) or composite response elements which bind receptors in addition to other transcription factors (Diamond et al, 1990). However, it is increasingly proposed that such events proceed most efficiently in an appropriate growth factor environment, with steroid hormone and growth factor signalling pathways 'cross-talking' to reinforce each others' signalling. While many of the relevant growth factors and their receptors are expressed by the breast cancer epithelial cells themselves, thereby potentially working in an autocrine manner, additional paracrine factors may be liberated from the surrounding stroma. In each instance, several potential points of interaction between steroid hormone and growth factor signalling pathways have been identified. A number of these are detailed below and are illustrated in Figure 1.

### *The ER is a target for growth factor-induced kinase activity (Figure 1.1)*

Numerous studies have now shown that the ER protein is subject to phosphorylation and activation by several peptide growth factors (e.g. IGF1 [Aronica and Katzenellenbogen, 1993], EGF, transforming growth factor-α [TGF-α, Bunone et al, 1996] and heregulin [Pietras et al, 1995]), events which can subsequently initiate ERE-mediated gene expression (Ignar-Trowbridge et al, 1996; Lee et al, 1997). These events are believed to be effected by

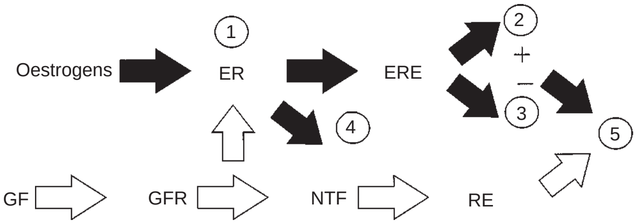

**Figure 1** Cross-talk between ER and growth factor signalling pathways. ER, oestrogen receptor; ERE, oestrogen response element; GF, growth factor; GFR, growth factor receptor; NTF, nuclear transcription factor; RE, response element downstream signal transduction molecules such as MAP kinase, which has been shown to activate ER possibly by a direct phosphorylation of serine 118 located in the A/B region of the ER (Kato et al, 1995). Additional transduction molecules demonstrated to target the ER to date include casein kinase II, pp90rsk1, protein kinase C δ, cyclin A/cdk2, Rho pathway elements and p60c-src (Ali et al, 1993; Arnold et al, 1994, 1997; Le Goff et al, 1994; Casalini et al, 1997; Trowbridge et al, 1997; Zwijsen et al, 1997; Joel et al, 1998; Lahooti et al, 1998; Rubino et al, 1998; Tzahar and Yarden, 1998). Significantly, growth factors and downstream signal transduction pathways appear to differentiallyregulate the two transcriptional activator functions of the ER (i.e. AF-1 and AF-2), with the former being more responsive to EGF, TGF-α and mitogen activated protein (MAP) kinase signalling (Bunone et al, 1996). While activation by these factors occurs most efficiently in the presence of oestrogens, their promotion of AF-1 and AF-2 responses certainly appears adequate for initiating transcription in the absence of the steroid hormone. An increasing number of additional cell signalling pathways appear to also impact on the bioactivity of ER, including the pineal hormone melatonin (Ram et al, 1995), neurotransmitters such as dopamine (Gangolli et al, 1997), and second messengers including cAMP (Cho and Katzenellenbogen, 1993). An emerging concept for steroid hormone receptors is therefore that they function not only as direct transducers of steroid hormone effects but, as members of the cellular nuclear transcription factor pool, also serve as key points of convergence for multiple signal transduction pathways (McDonnell et al, 1995).

### *Oestrogens stimulate positive elements of growth factor signalling pathways, including cell attachment factors which may facilitate growth factor-directed cell proliferation (Figure 1.2)*

Oestrogen sensitivity and endocrine response have been extensively investigated in experimental models of human breast cancer both in vitro and in vivo. Based on these studies (Gee et al, 1996; Nicholson et al, 1996), it is becoming increasingly evident that oestrogens can promote the autocrine expression of growth factor signalling pathway components (Figure 1.2a), notably TGF-α (Bates et al, 1988), IGF-II (Brunner et al, 1993) and growth factor receptors (e.g. epidermal growth factor receptor [EGFR; Berthois et al, 1989] and IGF-IR [Freiss et al, 1990]), in oestrogen-responsive (MCF-7 and T47-D) and oestrogen-dependent (ZR-75-1) human breast cancer cell lines. In the latter instance, the IGF-IR has also been shown to be activated by oestrogen (Richards et al, 1996; Guvakova and Surmacz, 1997), subsequently recruiting downstream signalling components, notably including insulin receptor substrate-1 (IRS-1; Richards et al, 1996; Guvakova and Surmacz, 1997), which in turn may be oestrogen-regulated (Westley et al, 1998). Such actions, which are often antagonized by anti-oestrogens (Gee et al, 1996; Nicholson et al, 1996), could significantly supplement the cellular growth responses directly primed by oestrogens (Cho and Katzenellenbogen, 1993; Smith et al, 1993). In addition, it appears that oestrogens directly stimulate (while anti-oestrogens inhibit) the tyrosine kinase activities both of the EGFR-related protein c-*erb*B-2 (Matsuda et al, 1993) and of c-src (Migliaccio et al, 1993), the activation of which can provide important mitogenic signals to epithelial cells (Figure 1.2b) through the recruitment of the p21ras/Raf/MAP kinase pathway (James et al, 1994; Troppmain et al, 1994).

Commonly, the frequency with which a cell divides in vitro is dependent upon its adherence, increasing as cells spread out over the extracellular matrix. This may not only facilitate increased nutrient uptake, but also the ability of the cell to capture growth factors, this being particularly evident at focal adhesion contacts which function as sites for priming of intracellular signals (Weisberg et al, 1997). In this light, oestrogens in addition to stimulating growth factor signalling pathways directly, can promote cell–cell and cell–matrix adhesion (Millon et al, 1989; De Pasquale, 1998), thereby facilitating growth factor directed cell proliferation. Oestrogens have thus been shown to induce laminin receptor, together with various extracellular matrix components and cell membrane adhesion proteins (Castronovo et al, 1989), events which may be blocked by anti-oestrogens (Millon et al, 1989). Indeed, the anti-oestrogen toremifene has been shown to inhibit the phorbol ester enhanced $\alpha_2 \beta_1$ integrin-dependent adhesion of MCF-7 breast carcinoma cells (Maemura et al, 1995).

### *Oestrogens inhibit negative elements of growth factor signalling pathways (Figure 1.3)*

As well as the positive influences exerted by oestrogens on growth factor signalling pathways detailed above, it is notable that in parallel they diminish (while anti-oestrogens induce) the expression of the growth inhibitory factor TGF-β (Knabbe et al, 1987) in several oestrogen-responsive human breast cancer cell lines. Oestrogens thus serve to inhibit the expression of a factor implicated in the induction of programmed cell death (Perry et al, 1995) and which acts through the p38/Jun kinase (JNK) pathway (Hill, 1996).

Additionally, however, it is of particular significance that oestrogens have been reported to inhibit expression of tyrosine phosphatases in ER-positive breast cancer cells to increase growth factor mitogenic activity, while both steroidal and non-steroidal anti-oestrogens increase phosphatase activity (Freiss and Vignon, 1994; Freiss et al, 1998). Tamoxifen, for example, inhibits the mitogenic activity of EGF by promoting significant dephosphorylation of EGFR, an effect believed to be ER-mediated (Freiss et al, 1990; Freiss and Vignon, 1989). It appears that such EGFR-dephosphorylation is accomplished via an increase in tyrosine phosphatase activity, as evidenced not only by an effective inhibition by sodium orthovandate (a broad-spectrum phosphatase inhibitor), but furthermore by a time- and dose-dependent increase in membrane phosphatase activity with the anti-oestrogen (Freiss and Vignon, 1998). In this light, two tyrosine phosphatases have been identified which appear to be regulated by oestrogens and anti-oestrogens, LAR and FAP-1 respectively (Freiss et al, 1998).

 © 2000 Cancer Research Campaign

Significantly, antisense inhibition of FAP-1 expression abolishes the anti-oestrogen-mediated inhibition of growth factor mitogenic activity, although the 'pure' anti-oestrogen ICI 182,780 appears to retain inhibitory activity under these conditions suggesting that the effects of this compound are FAP-1-independent (Freiss et al, 1998).

### *The ER interacts with growth factor-induced nuclear transcription factors, co-activators/co-repressors and additional proteins to target a diversity of response elements (Figure 1.4)*

An important feature of growth factor signalling is its potential to activate several profiles of nuclear transcription factors which subsequently serve to promote the expression of genes participating in a diversity of end points, including cell cycle progression. For example, in addition to its phosphorylation of the ER protein, growth factor-induced MAP kinase (ERK1/2) directly activates Elk-1/p62TCF (Gille et al, 1995). This latter transcription factor subsequently forms a ternary complex with p67SRF (serum response factor) and primes Fos expression via the c-*fos* serum response element (Gille et al, 1995). Similarly, JNK (also a member of the MAP kinase family [Paul et al, 1997; Lewis et al, 1998]) phosphorylates the c-Jun protein which subsequently heterodimerizes with Fos (Minden et al, 1994). The resultant complex, AP-1, is of central importance since it directly targets the 12-*O* tetradecanoyl-phorbol-13 acetate-responsive element (TPA-RE), a sequence found in the promoters of many genes involved in a plethora of cellular end points, including proliferation and survival (Pfahl, 1993).

In this light, it has been reported that oestrogens can significantly enhance growth factor induced AP-1 activity in hormone sensitive breast cancer cells (Phillips et al, 1993). This feature is believed to be a consequence of productive protein–protein interactions between the ER and the AP-1 complex (Rochefort, 1995), a phenomoneon also recently demonstrated to occur between ER and the transcription factor SP-1 (Porter et al, 1997; Duan et al, 1998; Sun et al, 1998; Xie et al, 1999). Thus, ER appears able to activate genes containing AP-1 sites in their promoters (Webb et al, 1995), providing a mechanism whereby ER signalling may be markedly diversified. Initial studies suggested that anti-oestrogens antagonized growth factor induced AP-1 activity, with maximal inhibition by pure anti-oestrogens (Phillips et al, 1993). However, subsequent investigations (albeit performed in uterine cells) have suggested that the tamoxifen–ER complex may also act agonistically on promoters regulated by the AP-1 site (Webb et al, 1995). In contrast to the above, ER may repress the activity of the transcription factor NF-κB (Nakshatri et al, 1997), which regulates expression of many cytokines (such as IL-6) and growth factors (Sharma and Narayanan, 1996). αER-dependent inhibition of IL-6 again appears to be mediated via a direct protein–protein interaction with NF-κB (Ray et al, 1997).

Finally, it should be remembered that ER/ERE-mediated gene transcription is also significantly enhanced by the recruitment of several co-activators and/or by overcoming the effects of co-repressor proteins (McDonnell et al, 1992) that may feasibly be regulated by growth factor signal transduction pathways (Hanstein et al, 1996; Smith et al, 1996). Indeed, an increasing number of co-activators and co-repressors that can interact with the ER have been described (Parker, 1998), including the co-activators SRC-1, RIP-140 and AIB1 (Anzick et al, 1997; Smith et al, 1997; Parker, 1998), and the co-repressors Ssn6 and SMRT (McDonnell et al, 1992; Smith et al, 1997; Lavinsky et al, 1998). Of particular interest is the co-activator CREB-binding protein (CBP)/p300, which is believed to be a component of multiple signalling pathways including cAMP signal transduction (Hanstein et al, 1996; Smith et al, 1996). Additional proteins also under growth factor regulation have been shown to interract with the ER including the cell cycle protein cyclin D1 (Lavoie et al, 1996). This protein can activate ER by direct binding, as well as by recruiting co-activators of the SRC-1 family to the ER (Zurijsen et al, 1997, 1998).

### *Steroid hormone and growth factor signalling pathways influence common growth regulatory genes (Figure 1.5)*

In order for cells to proliferate, they initially need to be recruited into the cell cycle and then be induced to progress through it. These outcomes are orchestrated by at least two series of events which can be jointly influenced by steroid hormone and growth factor signalling pathways (Musgrove et al, 1993; Prall et al, 1998): firstly, the induction of intermediate early response genes, such as c-*fos* (Morishita et al, 1995; Duan et al, 1998), c-*jun* (Morishita et al, 1995; Mohamood et al, 1997) and c-*myc* (Dubik and Shiu, 1992; Musgrove et al, 1993), and secondly, the regulation of G1 cyclins (e.g. cyclin D1) and their partner kinases and inhibitors which are involved in restriction point control (Musgrove et al, 1993; Lukas et al, 1996). Joint activation of these pathways by oestrogens and growth factors would at a minimum reinforce mitogenic signals to responsive cells, and might even result in synergistic interactions between overlapping elements. Additionally, it is likely that steroid hormones (Kyprianou et al, 1991) and many growth factors (Amundadottir et al, 1996; Werner et al, 1997; Wang et al, 1998) influence the expression of cell survival factors in endocrine responsive cells, such as the bcl-2 protein (Huang et al, 1997; Wang et al, 1998).

### Changes in the tumour cell phenotype potentially perturb 'cross-talk' between oestrogen and growth factor signalling pathways in endocrine unresponsive disease (Figure 2)

The above data generated largely from model systems provides compelling evidence that many points of convergence exist for oestrogen- and growth factor-mediated signalling pathways, and that it is likely that growth responses in endocrine responsive breast cancers hence proceed more efficiently in a mixed oestrogen and growth factor milieu. In such tumours, although reductions in input signals from steroid hormones appear sufficient to promote extensive tumour remissions, there is an increasing body of evidence to suggest that phenotypic changes which would severely perturb the balance of steroid hormone and growth factor

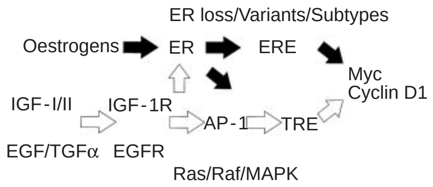

**Figure 2** Changes in breast cancer phenotype which may influence endocrine response

'cross-talk' may underlie the phenomenon of in vivo endocrine unresponsiveness in breast cancer.

### *EGFR and other members of the erbB receptor tyrosine kinase family*

Clinical data emerging in the late 1980s and early 1990s has convincingly shown a significant inverse relationship between the expression of the EGFR (reviewed in Klijn et al, 1992; Nicholson et al, 1994) and endocrine sensitivity in breast cancer. Thus, while patients whose tumours express low levels of EGFR frequently benefit from antihormonal drugs such as tamoxifen, women whose tumours express unusually high numbers of binding sites for EGF/TGF-α (Nicholson et al, 1989) or significant cell membrane-associated EGFR immunostaining (Nicholson et al, 1994) are largely de novo endocrine unresponsive. Although to some degree these associations may be simply explained by the inverse relationship known to exist between the oestrogen and EGFR, with ER negativity thus being commonly associated with EGFR positivity, nevertheless a direct involvement of the EGFR in growth responses in endocrine unresponsive disease has been suggested, with increased EGFR levels directly correlating both with elevated tumour proliferation and poor prognosis (Nicholson et al, 1997*a*, 1997*b*). Such a growth input would be likely to be pivotal to ER-negative/EGFR-positive tumours, since their lack of steroid hormone receptor expression would obviously preclude steroid hormone receptor mitogenic signalling. In addition, such an input might also be important to the proportion of de novo resistant ER-positive tumours maintaining elevated EGFR expression, since the absence of second-line responses in such patients similarly indicates a dislocation from steroid hormone receptor signalling.

Importantly, the inverse association between ER and EGFR also occurs at a cellular level (Sharma et al, 1994*b*, 1994*c*), where the long-term action of oestrogen is to suppress the expression of the EGFR (Berthois et al, 1989). In this light, it has been suggested that antihormonal measures which deprive breast cancer cells of oestrogens may consequently encourage increased cellular expression of the EGFR, a phenomenon perhaps culminating in the development of an acquired endocrine-resistant phenotype deriving an increased growth stimulus from EGFR signalling. Interestingly, this is a common phenotypic feature of endocrine-resistant breast cancer cells generated in vitro following either long-term exposure to anti-oestrogens or prolonged oestrogen deprivation (Gee et al, 1996; Nicholson and Gee, 1996), as exemplified by our own 'in house' acquired resistant MCF-7 sub-lines. Surprisingly, clinical and experimental data would suggest that such cells rarely lose all ER expression in parallel (Gee et al, 1996; Nicholson and Gee, 1996; Robertson, 1996), and as such the tumour re-growth hallmarking antihormonal relapse must be biologically distinct from the 20–30% of tumours displaying an ER-negative/EGFR-positive endocrine unresponsive phenotype at the time of clinical presentation (Nicholson et al, 1997*a*,*b*).

Tumour expression of an additional erbB family member, the c-*erb*B2 protein, has similarly been associated with endocrine unresponsiveness (Nicholson et al, 1993). Indeed, tumour co-expression of both EGFR and c-erbB2 appears to be associated with particularly aggressive phenotypes which lead to poor prognosis and resistance to endocrine treatment (Nicholson et al, 1997*a*, 1997*b*). This may be a direct result of the formation of heterodimeric receptor complexes which are highly efficient in their transmittance of mitogenic signals, conferring a cellular ability to escape the growth restraints exerted by hormonal therapy. In contrast, however, although interactions between either EGFR or c-*erb*B2 and an additional family member c-*erb*B3 synergistically enhance their mitogenic and transforming activity on 3T3 fibroblast cells in vitro (Alimandi et al, 1995; Wallasch et al, 1995; Tzahar et al, 1996), readily detectable levels of c-*erb*B3 (and c-*erb*B4) are surprisingly more frequent in well-differentiated ER-positive endocrine responsive clinical breast cancer, where EGFR (and often c-*erb*B2) expression is at its lowest (Knowlden et al, 1998). Patterns of expression (and therefore potentially the heterodimeric interactions) of the erbB family members thus appear to vary dramatically between hormone-sensitive and de novo insensitive disease. Given the increased expression of EGFR and the resultant overt sensitivity to an EGFR selective tyrosine kinase inhibitor, ZM 1839 exhibited by our MCF-7 cells emergent either following long-term antihormonal exposure or prolonged oestrogen deprivation, it is similarly likely that changes in these receptor patterns are a general feature of the acquired endocrine resistant state (McClelland and Nicholson, in preparation). Importantly, however, second-line endocrine responses occur in many acquired resistant breast cancer patients. Moreover, there is significant expression of the steroid hormone receptor at relapse, and in vitro inhibitory studies with the pure anti-oestrogen ICI 182780 confirm that ER is functional and actively contributory towards acquired tamoxifen- and oestrogen-resistant breast cancer growth. These data thus identify a maintained importance for ER (and hence potentially 'cross-talk') in acquired resistant growth that is also potentially EGFR-driven.

### *TGF-α and other* erb*B receptor ligands*

Enhanced production of TGF-α has been observed in transformed rodent and human fibroblast and epithelial cells, where it may function as a downstream intermediary in the transformation pathway elicited by oncogenes (Salomon et al, 1990). It has been suggested that TGF-α may act to induce hyperplastic responses in transformed breast cells, and thereby act as a promotional agent in combination with a normal background of mutational events (Matsui et al, 1990; Sandgren et al, 1990). Certainly, TGF-α has been demonstrated to be present in readily detectable amounts in clinical breast cancer specimens (Ciadello et al, 199; Lundy et al, 1991; Umekita et al, 1992), where its increased expression has been related to primary endocrine insensitivity in ER-positive disease (Nicholson et al, 1994), possibily through substantial ligand-independent activation of the ER as noted to occur experimentally. Furthermore, our recent examination of sequential clinical breast cancer biopsy specimens obtained during tamoxifen treatment is also supportive of elevated TGF-α protein expression being involved in acquired endocrine resistance in ER-positive disease, while diminished expression appears to be a therapeutic feature of those patients exhibiting a good quality and longer duration of initial response (Gee et al, in preparation). Although to date no direct associations have been reported between the cellular levels of TGF-α and ER/ERE-mediated events in vivo, ER-positive and ER-negative tumours with elevated cellular levels of TGF-α show an increased growth fraction, as monitored with the Ki-67 antibody (Nicholson et al, 1994, 1997*a*, 1997*b*). These data certainly suggest that elevated expression of this growth factor may comprise an integral part of the driving force behind the growth of many breast cancers, or may at least confer a significant growth or survival advantage upon such cells (Nicholson et al, 1994, 1999*a*, 1999*b*).

 *© 2000 Cancer Research Campaign*

Although no published in vivo data exists which relates the cellular levels of other ligands for the *erb*B family of receptor tyrosine kinases and endocrine response, multiple studies have shown that breast tumours express variable amounts of EGF and amphiregulin (ligands for the EGFR), together with heregulin α, β and γ and betacellulin (ligands for c-*erb*B3/4 [Lupu et al, 1995, 1996]). In this light, our preliminary examination of relevant ligands for the *erb*B family has indicated that expression of the mRNA for heregulin β1 (reported to be the most potent ligand for the c-*erb*B3 receptor) is associated with ER and c-*erb*B3 positivity in well-differentiated tumour types and is inversely associated with EGFR (Knowlden et al, in preparation). ErbB signalling in well-differentiated tumour cells would appear, therefore, biased towards c-*erb*B3/4, while anomalous increased expression of TGF-α/EGFR/c-*erb*B-2 in endocrine-insensitive disease may direct tumour growth response pathways away from their strict reliance on oestrogens, possibly towards substantial ligand-independent activation of the ER.

### IGF family

Many clinical breast carcinomas contain membrane-bound receptors for IGFs, and their ligands, IGF-I and IGF-II, are generally more potent mitogens for human breast cancer cells than either TGF-α or EGF (Gee et al, 1996, Nicholson et al, 1999*a*). Indeed, there is an increasing body of evidence demonstrating that IGF signalling plays a significant role in growth and survival of endocrine-responsive cells particularly under steroid-rich conditions (Arteaga et al, 1989), where synergistic interactions with oestrogens have been reported (Westley et al, 1989). Additionally, both IGF-I and IGF-II reputably influence the in vitro expression of oestrogen-regulated genes such as PR (Cho et al, 1994, Giani et al, 1998), Fos (Wosikowski et al, 1992) and the novel oestrogen-regulated gene pLIVI (El-Tanani and Green, 1997*b*), a gene that may have a role in directing metastatic spread in ER-positive disease (Manning et al, 1993, 1995). Although little is directly known about the IGF receptor family and their influence on clinical responsiveness of breast cancer to endocrine treatments, a correlation has been reported between IGFR-I expression and better clinical outcome (Papa et al, 1993). Potentially, therefore, such tumours exhibiting productive interactions between ER and IGF signalling may be particularly growth-sensitive to steroid hormone withdrawal.

Interestingly, acquired resistance to tamoxifen in vitro is reported to be accompanied by substantial increases in IGF-I binding in MCF-7 cells (Wiseman et al, 1993), while marked overexpression of IGFR in vitro reduces oestrogen growth requirements (Guvakova and Surmacz, 1997). Given the synergism apparent between IGF and oestrogen signalling pathways, such increases in the IGFR may serve to substantially enhance the partial oestrogenicity which is a feature of tamoxifen, ultimately allowing tumour cell re-growth during therapy. Similar changes occuring in vivo in elements of the IGF signalling pathway may feasibly contribute towards the acquired resistance phenomenon in the clinic. Additional elements which may similarly influence the relationship between endocrine response and IGFs include the presence or absence of specific binding proteins (IGFBPs) which are known to enhance or suppress IGF signalling. Breast cancer cells have been found to express several IGF-binding proteins, some of which are regulated by oestrogens and anti-oestrogens (Yee, 1998).

### Intracellular components of growth factor signalling pathways

In clinical specimens, Sivaraman et al (1997) demonstrated that hyperexpression of MAP kinase is a feature of clinical breast cancer. In this light, our own recent studies using antibodies which detect fully-activated erk1/2 MAP kinase have shown a highly significant relationship between increased activation and a poorer quality and shorter duration of response to the anti-oestrogen tamoxifen, as well as with a reduced survival time in ER-positive patients, while substantial increases also occur at the time of disease relapse (Gee et al, submitted). As stated above, MAP kinase has been shown, in addition to its inherent capacity to influence AP-1 and Elk-1 signalling, to activate the ER possibly by direct phosphorylation on Ser-118 located in the A/B region of the ER (Kato et al, 1995). Since this region contains the ligand independent AF-1 domain (Tzukerman et al, 1994), it remains a possibility that increased levels of activated erk1/2 MAP kinase may contribute substantially to growth responses via AF-1 driven transcriptional events originating from unoccupied or indeed anti-oestrogen-occupied ER.

Elevated levels and/or activity of additional intracellular molecules comprising growth factor signalling pathways have also been noted in malignant breast, including pp60c-src (Lehrer et al, 1989), Grb2 (Daly et al, 1994), RHAMM (Wang et al, 1998), Ras (Dati et al, 1991; Archer et al, 1995), Raf (Callans et al, 1995) and protein kinase C (PKC) (Gordge et al, 1996). Importantly, in a number of instances, overexpression of such components in ER-positive, hormone-sensitive breast cancer in vitro following transfection of appropriate vectors leads to an altered sensitivity to hormones and antihormonal agents (Van Roy et al, 1990; El-Ashry et al, 1997). Although the mechanisms underlying these artificially acquired changes in endocrine response have not been fully documented, it is notable that both PKCδ and c-src have, like MAP kinase, have been suggested to target the ER, phosphorylating Ser-122 (Lahooti et al, 1998) and Y-537 respectively (Arnold et al, 1997). Such interactions once again raise the possibility that growth factor-induced kinase activity, in addition to directly signalling onto specific nuclear transcription factor end points, may alter the behaviour of the ER protein under oestrogen-deprived or antihormone-occupied conditions, thereby generating resistance to endocrine measures. Antihormone resistance, therefore, may arise from altered ER phosphorylation patterns influencing its transcriptional activation.

### Nuclear transcription factors

As previously stated, an important element in growth factor-induced cell proliferation is the induction and activation of the AP-1 complex (Davis, 1995) and elevated expression of AP-1 activity has been observed in some human breast tumours, as compared to normal adjacent tissue (Linardopoulos et al, 1990). The Jun component of AP-1 is thus reported to be elevated in breast cancer (Tinrakos et al, 1994), and importantly there is an increasing body of in vitro and in vivo evidence to implicate the nuclear transcription factor Fos in the control of many processes associated with the ER-positive neoplastic breast cell, most notably in its acquisition of endocrine independency and invasive capabilities (Gee et al, 1995). Thus, we have demonstrated significant associations between elevated Fos protein expression and increased proliferation, de novo endocrine insensitivity (Gee et al, 1995) and furthermore a worsened patient outlook in clinical breast cancer (Gee et

© 2000 Cancer Research Campaign 

al, 1995), also noted by Bland et al (1995). Furthermore, our recent examination of sequential clinical breast cancer biopsy specimens obtained during tamoxifen treatment is also supportive of elevated Fos protein expression being involved in both primary and ER-positive acquired endocrine resistance (Gee et al, 1999), while diminished Fos expression appears to be a therapeutic feature of patients with a good quality and longer duration of response. Our clinical findings demonstrating therapeutic increases in the Fos component of the AP-1 complex associated with endocrine resistance are mirrored by limited in vitro studies. As such increased AP-1 DNA binding activity has been observed to be a feature of tamoxifen-resistant ER-positive breast cancer cells in vitro (Dumont et al, 1996), whilst prolonged tamoxifen exposure appears to render this anti-oestrogen agonistic in such cells via its augmentation of the phorbol ester-inducible expression of a chimeric AP-1 response (Astruc et al, 1995; Badia et al, 1995). These studies clearly reveal the importance of AP-1/ER signalling in directing long-term cellular responses to tamoxifen and its agonistic/antagonistic profile.

Sadly, little is known about the relationship between ER and additional nuclear transcription factors during the development of either endocrine insensitivity or acquired resistance. NF-κB/Rel is present in increased amounts in a proportion of clinical breast cancer specimens (Dejardin et al, 1995; Sovak et al, 1997) and has been linked to tumour progression in vitro (Nakshatri et al, 1997). Indeed, its increased expression in two human breast cancer cell lines has been suggested to lead to an inhibition of apoptosis (Sovak et al, 1997). Expression of Myb (a transcription factor that has been linked with cell cycle progression and appears to positively influence the expression of cyclin D1 [Sala and Calabretta, 1992; IGF-1 [Reiss et al, 1991] and bcl-2 [Thompson et al, 1998]) is commonly increased in ER-positive disease (Guerin et al, 1990; Gudas et al, 1995). Finally, the Ets-related transcription factor PEA3, a nuclear transcription factor primed by c-*erb*B2, appears increased in tumours overexpressing this receptor and moreover relates to progression in breast cancer (Benz et al, 1997). Growth factor-directed/constitutive expression of these factors may thus serve to influence endocrine response.

### Negative elements of growth factor signalling pathways

TGF-β is the most potent known inhibitor of the progression of normal mammary epithelial cells through the cell cycle (Reiss and Barcellos-Hoff, 1997). In clinical breast cancer, TGF-β proteins (Walker and Deering, 1992) or mRNAs (MacCallum et al, 1994) are present in many samples examined, usually at significantly higher levels than observed in the normal breast, indicating that such cancers may often be growth-refractory to the inhibitory activity of this factor (Travers et al, 1988, Reiss and Barcellos-Hoff, 1997). It is notable, however, that the levels and patterns of expression of TGF-β1–3 are highly variable (MacCallum et al, 1994).

In keeping with the reported effects of this growth factor on the extracellular matrix (Reiss and Barcellos-Hoff, 1997), several studies have indicated a positive relationship between TGF-β1 and both disease progression (Gorsch et al, 1992) and lymph node metastasis (Walker and Deering, 1992; Reiss and Barcellos-Hoff, 1997), with TGF-β1 localizing to the advancing epithelial edge of primary tumours and lymph node metastases (Dalal et al, 1993). Similarly, the detection of all three isoforms of TGF-β mRNA in breast cancer specimens is associated with lymph node involvement (MacCallum et al, 1994; Reiss and Barcellos-Hoff, 1997), with TGF-β1 mRNA levels being highest in ER-positive disease (Amoils and Bezwoda, 1997).

Although the relationship between TGF-β and endocrine sensitivity of breast cancer has not been been studied in great depth in clinical breast cancer, an early study was performed on 11 patients who had received tamoxifen for 3–6 months prior to surgery (Thompson et al, 1991). Unexpectedly high levels of TGF-β1 mRNA were found in patients whose tumours increased in size and were unresponsive to the anti-oestrogen. It is possible that progression during tamoxifen therapy may thus be due to a failure of the autocrine inhibitory functions of TGF-β1 either alone (as noted in in vitro [Herman and Katzenellenbogen, 1994]) or in combination with a paracrine stimulation of stromal cells or angiogenesis. Certainly, up-regulation of TGF-β1 mRNA in breast cancer cells in vitro following their transfection with either v-H-ras or TGF-β1 cDNA (Arteaga et al, 1993) leads to oestrogen growth-independence. Such cells, however, may also show parallel increases in TGF-α and IGF-1, together with a loss of growth response to insulin and bFGF (Daly et al, 1995). In contrast to the TGF-β1 clinical data, several studies have noted that the TGF-β2 isoform increases both in tumours and in plasma during tamoxifen therapy in responders, with no increases recorded in initial progressors (Knabbe et al, 1996; MacCallum et al, 1996). Interestingly, antibodies to TGF-β have been shown in a recent study to reverse tamoxifen resistance in LCC2 breast cancer cells (Arteaga et al, 1999), strongly implicating the pleotrophic properties of TGF-β in the development of this condition.

### Genetic events in growth factor expression and cell cycle control

Breast cancer cells, in common with other tumour types, are subject to genetic alterations, notably including those targeting growth factor-associated pathways and cell cycle control elements, and it is likely that such genetic changes would serve to markedly influence cellular response to their steroid hormone and anti-hormone environment (Dorssers and van Agthoven, 1996; Osin et al, 1998).

To date multiple activated oncogenes have been identified in breast cancer, together with the loss of several suppressor gene activities (Walker et al, 1997). These include an amplification of the c-*erb*B2 oncogene (Seshadri et al, 1993; Ross and Fletcher, 1998), which potentially directly alters the balance of growth factor signalling through the *erb*B family of receptor tyrosine kinases (see 'The ER is a target for growth factor-induced kinase activity'), and elevated expression of the Ras oncogene (Dati et al, 1991; Watson et al, 1991), which in culture not only increases the cellular output of several autocrine growth factors, but also serves to activate the ras/raf/MAP kinase signalling cascade (Janes et al, 1994). Altered signal transduction in such cells would thus serve to promote an increased expression and activity of multiple nuclear transcription families (Gille et al, 1995; Whitmarsh et al, 1996; Wasylyk et al, 1998), potentially including the steroid hormone receptors themselves (Kato et al, 1995).

Significantly, c-*myc* is also overexpressed in many breast tumours, where it relates to an increased proliferative activity, elevated tumour grade and disease spread to unfavourable sites (Kreipe et al, 1993). In our own unpublished series, Myc expression is particularly prominent within ER-positive de novo progressive disease. Although the precise molecular mechanisms which

 © 2000 Cancer Research Campaign lead to such elevated expression of Myc remain to be established, it is certainly interesting that Myc expression in clinical material correlates with that of TGF-α and activated erk 1/2 MAP kinase. Indeed, both TGF-α and MAP kinase are signalling parameters which have been shown to impinge on and synergize with Myc in the control of proliferation in many cancers both in vivo and in vitro (Amundadottir et al, 1996; Gupta and Davis, 1994; Nass and Dickson, 1998; Santoni-Rugiu et al, 1998). Additionally, TGF-α has also been reported to be a survival factor for mammary tumour cells that overexpress Myc, thereby potentially enabling increased Myc-directed cell proliferation to occur (Nass et al, 1996), while limiting any apoptosis-inducing activity known to be an additional feature of this nuclear transcription factor (Amundadottir et al, 1996). Since Myc has been shown to mimic the effects of oestradiol in promoting S phase entry (Prall et al, 1998), it is certainly feasible that Myc expression, together with other altered elements of growth factor signalling, may be of considerable importance in modifying endocrine response.

Additionally, our recent collaborative studies performed with Professor Robert Sutherland (Garvan Institute, Sydney) have demonstrated that the proportion of breast cancers overexpressing the key cell cycle protein cyclin D1 is much greater than had previously been appreciated from gene amplification studies of the chromosome 11q13 locus, suggesting that aberrant transcriptional/translational regulation is relatively common within such tumours (Hui et al, 1996). In this light, while many ER-positive tumours certainly overexpress the mRNA coding for the cell cycle protein cyclin D1 (Buckley et al, 1993; Hui et al, 1996; Kenny et al, submitted), interestingly its elevated expression marks a shortened disease-free interval, decreased time to local recurrence and metastasis, and poor patient survival characteristics. Growth factors signalling via erk 1/2 MAP kinase (Lavoie et al, 1996) and Myc (Santoni-Rugui et al, 1998) appear to contribute (together with steroid hormones [Sutherland et al, 1995]) to the regulation of cyclin D1. An important element in this event may be the eukaryotic initiation factor 4E (eIF4E), which is involved in regulation of cyclin D1 expression (Flynn and Proud, 1996) and is controlled by Ras/MAP kinase (Flynn and Proud, 1996; Sunenberg and Gingras, 1998) and Myc signalling (Jones et al, 1996). In this light, it is interesting that eIF4E is similarly overexpressed in breast carcinoma (Sorrells et al, 1998), where its expression again relates to poor patient prognosis (Kerekatte et al, 1995; Li et al, 1997, 1998). It is certainly feasible that the elevated erk 1/2 (and/or Myc) activity frequently observed in ER-positive, progressive disease may contribute to the marked proliferative capacity associated with resistance via increased positive influences on cyclin D1. Indeed, overexpression of cyclin D1 in ER-positive breast cancer cells in vitro has been shown in one study to subsequently allow unrestricted passage through the cell cycle, which can confer a resistance to growth inhibition by antioestrogenic agents (Wilcken et al, 1997*b*).

Interestingly, TGF-α has been observed to dramatically enhance c-*myc*-induced hepatocarcinogenesis in a transgenic mouse model, with the resultant hyperproliferative responses being not only associated with raised cellular expression of cyclin D1, but also with significant changes in additional components of cell cycle regulation (e.g. intense Rb hyperphosphorylation and increased E2F activity [Santoni-Rugui et al, 1998]). Clearly, aberrations in growth factor signalling are likely to impinge on several key growth/survival regulatory elements, thereby potentially influencing tumour growth and hence steroid hormone/antihormone response. Such positive effects on cell cycle progression may be further aided by the reduced expression of the downstream mediator of p53, p21/WAF-1, in many de novo endocrine-resistant patients (Nicholson et al, 1997*c*; McClelland et al, 1998).

Finally, BRCA1 expression may also play a role in influencing endocrine response in view of recent results that have shown that its mRNA levels are indirectly elevated in breast cancer cells in response to oestrogen (Spillman and Bowcock, 1996; Marks et al, 1997), while familial mutation associates with an endocrine unresponsive phenotype (Osin et al, 1998). Indeed, we have recently shown that BRCA1 and ER gene expression are closely associated in clinical breast cancer, where low levels of BRCA1 expression mark a propensity of the tumours to metastasize to distant sites (Seery et al, 1999).

### *ER loss, receptor variants/mutations and sub-types*

ER negativity is a relatively common event comprising some 20–30% of breast tumours at presentation, and is predictably associated with de novo endocrine resistance (Campbell et al, 1981; Nicholson et al, 1986, 1995; Merkel and Osborne, 1989; Robertson et al, 1992). Although the origins of the steroid hormone receptor-negative phenotype at presentation are as yet unknown (Ferguson and Davidson, 1997), TGF-α/EGFR/c-*erb*B2 signalling, and the intracellular transduction elements MAP kinase, PKC and AP-1, all appear of significance in relation to growth responses (Nicholson et al, 1997*a*, 1997*b*). Relevant mutations in the ER gene resulting in an inability to transcribe ER are likely to be extremely rare in breast cancer (Ferguson et al, 1998). However, a number of potential mechanisms preventing the efficient transcription or translation of the ER gene resulting in a lack of ER protein expression may exist. These mechanisms include: (i) transcriptional inactivation by hypermethylation of the CpG island in the regulatory region of the ER gene (Falette et al, 1990; Ottaviano et al, 1994; Lapidus et al, 1996); (ii) altered expression of transacting factors responsible for ER transcription (deConinck et al, 1995); and (iii) abnormalities in ER translation or synthesis of an unstable receptor protein (Ferguson and Davidson, 1997). Alternatively, ER-negative tumours may feasibly arise from the selective outgrowth of a sub-population of steroid receptor-negative cells which are likely to exist in the normal breast epithelium (Walker et al, 1991, 1992), although such selective outgrowth is reported to be very infrequent in vivo (Dowsett, 1996).

Although recent studies have revealed the ER protein may be subject to several mutations, as well as the generation of several truncated or exon deleted variant forms (Dowsett et al, 1997; Murphy et al, 1997) which theoretically may alter its functionality and ability to interact with growth factor signalling elements (Murphy et al, 1997), in practice the ER mutations and variants that have been noted in vivo are unlikely to provide a general mechanism for resistance to tamoxifen therapy in ER-positive disease (Karnik et al, 1994; Daffada et al, 1995). However, there may be a role in breast cancer for the relatively recently-identified ER sub-type (Dotzlaw et al, 1997; Leygul et al, 1998), ERβ, and its variants (Lu et al, 1998; Vladusic et al, 1998). In contrast to ERα (previously referred to as ER in the current text), wild-type ERβ promotes AP-1 activity in the presence of anti-oestrogens (Paech et al, 1997), while showing differential effects on ERE-mediated events (Watanabe et al, 1997; McInerney et al, 1998) and co-activator selectivity (Suen et al, 1998). Moreover, interactions between ERα/ERβ and other nuclear receptor interacting proteins which serve as co-activators and co-repressors of ER transcriptional activity may change during breast cancer progression and contribute to endocrine failure (Berns et al, 1998; Lavinsky et al, 1998). Unfortunately, although such proteins are often phosphorylated and thus potentially subject to growth factor-related kinase activation, virtually no clinical data is available in this subject area.

### Model of endocrine response and new therapeutic targets

Increasing knowledge of the molecular biology of ER and growth factor signalling is providing new ideas regarding the mechanisms of action of hormones and antihormones, and moreover possible explanatory hypotheses for the tumour growth associated with the phenomena of de novo and acquired endocrine resistance. A simplified working model for the transition of endocrine-responsive breast cancer to endocrine insensitivity/resistance has been compiled in summary of the data presented in this review.

In hormone-sensitive breast cancer cells, it is likely that external signals generated by steroid hormones and stimulatory growth factors act to induce/activate several classes of nuclear transcription factors (e.g. steroid hormone receptor, Fos, Jun, Myc, Elk-1 etc.). These influence patterns of gene expression leading to the gain of positive influences on cell cycle regulation (e.g. cyclin D1) and the suppression of negative influences (e.g. TGF-β). In the presence of adequate steroid hormone and growth factor input signals, cells are perceived to be recruited into the cell cycle and successfully progress through it. Equivalent pathways maintain cell survival. Although it is likely that cross-talk between steroid and growth factor pathways enables efficient growth signalling, reductions in the input signals originating from steroid hormones appear sufficient to reduce proliferation and induce programmed cell death, thereby leading to tumour remissions. In this model, differences between endocrine responses exhibited by normal and cancerous cells would be expected to be minimal if oncogenic events occurred in those cellular pathways which either act to limit the extent of growth, but still require an input signal for growth (i.e. which normally maintain tissue size and architecture through negative feedback and homeostasis mechanisms), or facilitate a more efficient use of input signals from steroid hormones.

In cancers unresponsive to current endocrine measures (Figure 3), we postulate that further alterations have occurred in those elements of growth factor signalling pathways which:

1. Have a positive influence on steroid hormone receptor signalling and which facilitate the biological functions of the receptor in a lowered endocrine environment (or indeed in the presence of antihormones which show oestrogen-like activity such as tamoxifen). Retention of the ER protein in such cells (as a continued orchestrator of growth responses) would facilitate additional responses to endocrine measures which act by different mechanisms (i.e. aromatase inhibitor/pure anti-oestrogen substituting for tamoxifen). Such second line responses certainly occur in over 50% of women with acquired resistant disease who have benefited from a first-line endocrine response.
2. Circumvent the cellular requirement for steroid hormones via by-passing those elements of their response pathways which impinge on cell proliferation and survival i.e. post-receptor mechanisms. Such phenotypic/genotypic changes may be severe enough to override the importance of ‘cross-talk’ and hence effectively dislocate growth from a reliance on the steroid hormone receptor. Importantly, the majority of patients who fail to respond to one form of endocrine therapy de novo rarely respond to another, suggesting that the influence of the ER in their tumour cells is entirely nullified or circumvented at the time of presentation. This mechanism may also account for the eventual development of acquired resistance to multiple endocrine measures.
3. Provide a mitogenic input for tumours lacking ER. ER negativity is predictably associated with de novo endocrine resistance, comprising ~20–30% of breast tumours at presentation. Although it is as yet unknown if such a phenotype arises from aberrant loss of the steroid hormone receptor or from selective outgrowth of steroid hormone receptor negative cells, the regulation of such tumours is severed from the steroid hormone environment and they appear wholly dependent on elements of growth factor signalling.

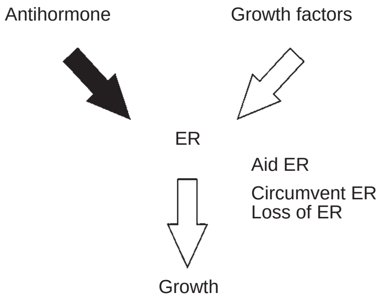

**Figure 3** Endocrine resistant disease

Based on the above model, it is clear that while the presence of ER within tumours obviously offers an opportunity for response to those antihormonal measures which directly target the ER, other elements of the complex breast cancer phenotype are likely to dictate the quality and duration of response and to be involved in relapse mechanisms. In the future, the identification of these elements is thus likely to be not only of great prognostic value in identifying those women likely to benefit from existing endocrine measures, but should promote the development of novel therapeutic strategies designed to delay the appearance of, treat, or even reverse endocrine resistance, thereby severely compromising the disease process (Nicholson et al, 1999*a*, 1999*b*). These might potentially include the targeting of:

i. Any continued use of steroid hormone receptor signalling
ii. Aberrant growth factor signalling
iii. Cross-talk between (i) and (ii)
iv. Genetic aberrations within additional growth regulatory components.

Although the fulfilment of these strategies will be a challenging goal for those involved in breast cancer research (Nicholson et al, 1999*a*, 1999*b*), success should significantly extend the value of endocrine measures and improve patient survival.

### ACKNOWLEDGEMENTS

The authors thank the Tenovus Charity for their continued support in this field.

 © 2000 Cancer Research Campaign

### REFERENCES

Ali S, Metzger D, Bornert JM and Chambon P (1993) Modulation of transcriptional activation by ligand-dependent phosphorylation of the human oestrogen receptor A/B region. *EMBO J* **12:** 1153–1160

Alimandi M, Romano A, Curia MC, Muraro R, Fedi P, Aaronson SA, Di Fiore PP and Kraus MH (1995) Cooperative signaling of ErbB3 and ErbB2 in neoplastic transformation and human mammary carcinomas. *Oncogene* **10**: 1813–1821

Amoils KD and Bezwoda WR (1997) TGF-beta 1 mRNA expression in clinical breast cancer and its relationship to ER mRNA expression. *Breast Cancer Res Treat* **42**: 95–101

Amundadottir LT, Nass SJ, Berchem GJ, Johnson MD and Dickson RB (1996) Cooperation of TGF alpha and c-Myc in mouse mammary tumorigenesis: coordinated stimulation of growth and suppression of apoptosis. *Oncogene* **13**: 757–765

Anzick SL, Kononen J, Walker RL, Azorsa DO, Tanner MM, Guan XY, Sauter G, Kallioniemi OP, Trent JM and Meltzer PS (1997) AIBI, a steroid receptor coactivator amplified in breast and ovarian cancer. *Science* **277**: 965–968

Archer SG, Eliopoulos A, Spandidos D, Barnes D, Ellis IO, Blamey RW, Nicholson RI and Robertson JFR (1995) Expression of ras p21, p53 and c-erbB-2 in advanced breast cancer and response to first line hormonal therapy. *Br J Cancer* **72**: 1259–1266

Arnold SF, Obourn JD, Jaffe H and Notides AC (1994) Serine 167 is the major estradiol-induced phosphorylation site on the human estrogen receptor. *Mol Endocrinol* **8**: 1208–1214

Arnold SF, Melamed M, Vorojeikina DP, Notides AC and Sasson S (1997) Estradiol-binding mechanism and binding capacity of the human estrogen receptor is regulated by tyrosine phosphorylation. *Mol Endocrinol* **11**: 48–53

Aronica SM and Katzenellenbogen BS (1993) Stimulation of estrogen receptor-mediated transcription and alteration in the phosphorylation state of the rat uterine estrogen receptor by estrogen, cyclic adenosine monophosphate, and insulin-like growth factor-I. *Mol Endocrinol* **7**: 743–752

Arteaga CL and Osborne CK (1989) Growth inhibition of human breast cancer cells in vitro with an antibody against the type I somatomedin receptor. *Cancer Res* **49(22)**: 6237–6241

Arteaga CL, Carty-Dugger T, Moses HL, Hurd SD and Pietenpol JA (1993) Transforming growth factor beta 1 can induce estrogen-independent tumorigenicity of human breast cancer cells in athymic mice. *Cell Growth Differ* **4**: 193–201

Arteaga CL, Koli KM, Dugger TC and Clarke R (1999) Reversal of tamoxifen resistance of human breast carcinomas in vivo by neutralizing antibodies to transforming growth factor-beta. *J Natl Cancer Inst* **91**: 46–53

Astruc ME, Chabret C, Bali P, Gagne D and Pons M (1995) Prolonged treatment of breast cancer cells with antiestrogens increases the activating protein-1-mediated response: involvement of the estrogen receptor. *Endocrinology* **136**: 824–832

Badia E, Duchesne MJ, Astruc M, Fuentes M, Gagne D, Nicolas JC, Pons M (1995) Modulation of cellular response expression during prolonged treatment with antiestrogens. *C R Seances Soc Biol Fil* **189**: 755–764

Bates SE, Davidson NE, Valverius EM, Freter CE, Dickson RB, Tam JP, Kudlow JE, Lippman ME and Salomon DS (1988) Expression of transforming growth factor-alpha and its mRNA in human breast cancer: its regulation by oestrogen and its possible functional significance. *Mol Endocrinol* **2**: 543–555

Benz CC, O'Hagan Rc, Richter B, Scott GK, Chang CH, Xiong X, Chew K, Ljung BM, Edgerton S, Thor A and Hassell JA (1997) HER2/Neu and the Ets transcription activator PEA3 are coordinately upregulated in human breast cancer. *Oncogene* **15**: 1513–1525

Berns EM, van Staveren IL, Klijn JG and Foekens JA (1998) Predictive value of SRC-1 for tamoxifen response of recurrent breast cancer. *Breast Cancer Res Treat* **48**: 87–92

Berthois Y, Dong XF and Martin PM (1989) Regulation of epidermal growth factor receptor by oestrogen and antioestrogen in the human breast cancer cell line MCF-7. *Biochem Biophys Res Commun* **159**: 126–131

Bland KI, Konstadoulakis MM, Vezeridis MP and Wanebo HJ (1995) Oncogene protein co-expression. Value of Ha-ras, c-myc, c-fos, and p53 as prognostic discriminants for breast carcinoma. *Ann Surg* **221**: 706–718

Bouzubar N, Walker KJ, Griffiths K, Ellis IO, Elston CW, Robertson JF, Blamey RW and Nicholson RI (1989) Ki-67 immunostaining in primary breast cancer: pathological and clinical associations. *Br J Cancer* **59**: 943–947

Brunner N, Yee D, Kern FG, Spang-Thomsen M, Lippman ME and Cullen KJ (1993) Effect of endocrine therapy on growth of T61 human breast cancer xenografts is directly correlated to a specific down-regulation of insulin-like growth factor II (IGF-II). *Eur J Cancer* **29**: 562–569

Buckley MF, Sweeney KJ, Hamilton JA, Sini RL, Manning DL, Nicholson RI, deFazio A, Watts CK, Musgrove EA and Sutherland RL (1993) Expression and amplification of cyclin genes in human breast cancer. *Oncogene* **8**: 2127–2133

Bunone G, Briand PA, Miksicek RJ and Picard D (1996) Activation of the unliganded estrogen receptor by EGF involves the MAP kinase pathway and direct phosphorylation. *EMBO J* **15**: 2174–2183

Callans LS, Naama H, Khandelwal M, Plotkin R and Jardines L (1995) Raf-1 protein expression in human breast cancer cells. *Ann Surg Oncol* **2**: 38–42

Campbell FC, Blamey RW, Elston CW, Morris AH, Nicholson RI, Griffiths K and Haybittle JL (1981) Quantitative oestradiol receptor values in primary breast cancer and response of metastases to endocrine therapy. *Lancet* **2**: 1317–1319

Casalini P, Menard S, Malandrin SM, Rigo CM, Colnaghi MI, Cultraro CM and Segal S (1997) Inhibition of tumorigenicity in lung adenocarcinoma cells by c-erbB-2 antisense expression. *Int J Cancer* **72**: 631–636

Castronovo V, Taraboletti G, Liotta LA and Sobel ME (1989) Modulation of laminin receptor expression by estrogen and progestins in human breast cancer cell lines. *J Natl Cancer Inst* **81**: 781–788

Cheung KL, Willsher PC, Pinder SE, Ellis IO, Elston CW, Nicholson RI, Blamey RW and Robertson JF (1997) Predictors of response to second-line endocrine therapy for breast cancer. *Breast Cancer Res Treat* **45**: 219–224

Cho H and Katzenellenbogen BS (1993) Synergistic activation of estrogen receptor-mediated transcription by estradiol and protein kinase activators. *Mol Endocrinol* **7**: 441–452

Cho H, Aronica SM and Katzenellenbogen BS (1994) Regulation of progesterone receptor gene expression in MCF-7 breast cancer cells: a comparison of the effects of cyclic adenosine 3′,5′-monophosphate, estradiol, insulin-like growth factor-I, and serum factors. *Endocrinology* **134**: 658–664

Ciardiello F, Kim N, McGeady ML, Liscia DS, Saeki T, Bianco C and Salomon DS. Expression of transforming growth factor alpha in breast cancer. *Ann Oncol* **2**: 169–182

Daffada AA, Johnston SR, Smith IE, Detre S, King N and Dowsett M (1995) Exon 5 deletion variant estrogen receptor messenger RNA expression in relation to tamoxifen resistance and progesterone receptor/pS2 status in human breast cancer. *Cancer Res* **55**: 288–293

Dalal BI, Keown PA and Greenberg AH (1993) Immunocytochemical localization of secreted transforming growth factor-beta 1 to the advancing edges of primary tumors and to lymph node metastases of human mammary carcinoma. *Am J Pathol* **143**: 381–389

Daly RJ, Binder MD and Sutherland RL (1994) Overexpression of the Grb2 gene in human breast cancer cell lines. *Oncogene* **9**: 2723–2727

Daly RJ, Carrick N and Darbre PD (1995) Progression to steroid autonomy is accompanied by altered sensitivity to growth factors in S115 mouse mammary tumour cells. *J Steroid Biochem Mol Biol* **54**: 21–29

Dati C, Muraca R, Tazartes O, Antoniotti S, Perroteau I, Giai M, Cortese P, Sismondi P, Saglio G and De Bortoli M (1991) c-erbB-2 and ras expression levels in breast cancer are correlated and show a co-operative association with unfavorable clinical outcome. *Int J Cancer* **47**: 833–838

Davis RJ (1995) Transcriptional regulation by MAP kinases. *Mol Reprod Dev* **42**: 459–467

deConinck EC, McPherson LA and Weigel RJ (1995) Transcriptional regulation of estrogen receptor in breast carcinomas. *Mol Cell Biol* **15**: 2191–2196

Dejardin E, Bonizzi G, Bellahcene A, Castronovo V, Merville MP and Bours V (1995) Highly-expressed p100/p52 (NFKB2) sequesters other NF-kappa B-related proteins in the cytoplasm of human breast cancer cells. *Oncogene* **11**: 1835–1841

DePasquale JA (1998) Cell matrix adhesions and localization of the vitronectin receptor in MCF-7 human mammary carcinoma cells. *Histochem Cell Biol* **110**: 485–494

Diamond MI, Miner JN, Yoshinaga SK and Yamamoto KR (1990) Transcription factor interactions: selectors of positive or negative regulation from a single DNA element. *Science* **249**: 1266–1272

Dorssers LC and van Agthoven T (1996) Genetic mechanisms of estrogen-independence in breast cancer. *Pathol Res Pract* **192**: 743–751

Dotzlaw H, Leygue E, Watson PH and Murphy LC (1997) Expression of estrogen receptor-beta in human breast tumors. *J Clin Endocrinol Metab* **82**: 2371–2374

Dowsett M (1996) Endocrine resistance in advanced breast cancer. *Acta Oncol* **35**: 91–95

Dowsett M, Daffada A, Chan CM and Johnston SR (1997) Oestrogen receptor mutants and variants in breast cancer. *Eur J Cancer* **33**: 1177–1183

Duan R, Porter W and Safe S (1998) Estrogen-induced c-fos protooncogene expression in MCF-7 human breast cancer cells: role of estrogen receptor Sp1 complex formation. *Endocrinology* **139**: 1981–1990

Dubik D and Shiu RP (1992) Mechanism of estrogen activation of c-myc oncogene expression. *Oncogene* **7**: 1587–1594

© 2000 Cancer Research Campaign 

Dumont JA, Bitonti AJ, Wallace CD, Baumann RJ, Cashman EA and Cross-Doersen DE (1996) Progression of MCF-7 breast cancer cells to antiestrogen-resistant phenotype is accompanied by elevated levels of AP-1 DNA-binding activity. *Cell Growth Differ* **7**: 351–359
El-Ashry D, Miller DL, Kharbanda S, Lippman ME and Kern FG (1997) Constitutive Raf-1 kinase activity in breast cancer cells induces both estrogen-independent growth and apoptosis. *Oncogene* **15**: 423–435
El-Tanani MK and Green CD (1997) Interaction between estradiol and growth factors in the regulation of specific gene expression in MCF-7 human breast cancer cells. *J Steroid Biochem Mol Biol* **60**: 269–276
Falette NS, Fuqua SA, Chamness GC, Cheah MS, Greene GL and McGuire WL (1990) Estrogen receptor gene methylation in human breast tumors. *Cancer Res* **50**: 3974–3978
Ferguson AT and Davidson NE (1997) Regulation of estrogen receptor alpha function in breast cancer. *Crit Rev Oncog* **8**: 29–46
Ferguson AT, Lapidus RG and Davidson NE (1998) The regulation of estrogen receptor expression and function in human breast cancer. In: *Biological and Hormonal Therapies of Cancer*, Foon KA and Muss HB (eds). Kluwer Academic Publishers: Boston
Flynn A and Proud G (1996) Insulin-stimulated phosphorylation of initiation factor 4E is mediated by the MAP kinase pathway. *FEBS Lett* **389**: 162–166
Freiss G, Prebois C, Rochefort H and Vignon F (1990) Anti-steroidal and anti-growth factor activities of anti-oestrogens. *J Steroid Biochem Mol Biol* **37**: 777–781
Freiss G and Vignon F (1994) Antiestrogens increase protein tyrosine phosphatase activity in human breast cancer cells. *Mol Endocrinol* **8**: 1389–1396
Freiss G, Puech C and Vignon F (1998) Extinction of insulin-like growth factor-I mitogenic signaling by antiestrogen-stimulated Fas-associated protein tyrosine phosphatase-1 in human breast cancer cells. *Mol Endocrinol* **12**: 568–579
Gangolli EA, Conneely OM and O'Malley BW (1997) Neurotransmitters activate the human estrogen receptor in a neuroblastoma cell line. *J Steroid Biochem Mol Biol* **61**: 1–9
Gee, JMW, Ellis IO, Robertson JFR, Willsher P, McClelland RA, Hewitt KN, Blamey RW and Nicholson RI (1995) Immunocytochemical localization of FOS protein in human breast cancers and its relationship to a series of prognostic markers & response to endocrine therapy. *Int J Cancer (Pred Oncol)* **64**: 269–273
Gee JWM, McClelland RA and Nicholson RI (1996) Growth factors and endocrine sensitivity in breast cancer. In: *Molecular and Clinical Endocrinology*, Pasqualini JR and Katzenellenbogen BS (eds), pp. 169–197. Marcel Dekker
Gee, JMW, Willsher P, Kenny FS, Robertson JFR, Pinder SE, Ellis IO and Nicholson RI (1999) Endocrine response and resistance in breast cancer: a role for the transcription factor FOS. *Int J Cancer (Pred Oncol)* **84**: 54–61
Giani C, Pinchera A, Rasmussen A, Fierabracci P, Bonacci R, Campini D, Bevilacqua G, Trock B, Lippman ME and Cullen KJ (1998) Stromal IGF-II messenger RNA in breast cancer: relationship with progesterone receptor expressed by malignant epithelial cells. *J Endocrinol Invest* **21**: 160–165
Gille H, Kortenjann M, Thomae O, Moomaw C, Slaughter C, Cobb MH and Shaw PE (1995) ERK phosphorylation potentiates Elk-1-mediated ternary complex formation and transactivation. *EMBO J* **14**: 951–962
Gorsch SM, Memoli VA, Stukel TA, Gold LI and Arrick BA (1992) Immunohistochemical staining for transforming growth factor beta 1 associates with disease progression in human breast cancer. *Cancer Res* **52**: 6949–6952
Gordge PC, Hulme MJ, Clegg RA and Miller WR (1996) Elevation of protein kinase A and protein kinase C activities in malignant as compared with normal human breast tissue. *Eur J Cancer* **32A**: 2120–2126
Gudas JM, Klein RC, Oka M and Cowan KH (1995) Posttranscriptional regulation of the c-myb proto-oncogene in estrogen receptor-positive breast cancer cells. *Clin Cancer Res* **1**: 235–243
Guerin M, Sheng ZM, Andrieu N and Riou G (1990) Strong association between c-myb and oestrogen-receptor expression in human breast cancer. *Oncogene* **5**: 131–135
Gupta S and Davis RJ (1994) MAP kinase binds to the NH2-terminal activation domain of C-Myc. FEBS Lett **353**: 281–285
Guvakova MA and Surmacz E (1997) Tamoxifen interferes with the insulin-like growth factor I receptor (IGF-IR) signaling pathway in breast cancer cells. *Cancer Res* **57**: 2606–2610
Hanstein B, Eckner R, DiRenzo J, Halachmi S, Liu H, Searcy B, Kurokawa R and Brown M (1996) p300 is a component of an estrogen receptor coactivator complex. *Proc Natl Acad Sci USA* **93**: 11540–11545
Herman ME and Katzenellenbogen BS (1994) Alterations in transforming growth factor-alpha and -beta production and cell responsiveness during the progression of MCF-7 human breast cancer cells to estrogen-autonomous growth. *Cancer Res* **54**: 5867–5874
Hill CS (1996) Signalling to the nucleus by members of the transforming growth factor-beta (TGF-beta) superfamily. *Cell Signal* **8**: 533–544
Huang Y, Ray S, Reed JC, Ibrado AM, Tang C, Nawabi A and Bhalla K (1997) Estrogen increases intracellular p26Bcl-2 to p21Bax ratios and inhibits taxol-induced apoptosis of human breast cancer MCF-7 cells. *Breast Cancer Res Treat* **42**: 73–81
Hui R, Cornish AL, McClelland RA, Robertson JFR, Blamey RW, Musgrove EA, Nicholson RI and Sutherland RL (1996) Cyclin D1 and estrogen receptor messenger RNA levels are positively correlated in primary breast cancer. *Clin Cancer Res* **2**: 923–928
Ignar-Trowbridge DM, Pimentel M, Parker MG, McLachlan JA and Korach KS (1996) Peptide growth factor cross-talk with the estrogen receptor requires the A/B domain and occurs independently of protein kinase C or estradiol. *Endocrinology* **137**: 1735–1744
Janes PW, Daly RJ, deFazio A and Sutherland RL (1994) Activation of the Ras signalling pathway in human breast cancer cells overexpressing erbB-2. *Oncogene* **9**: 3601–3608
Joel PB, Smith J, Sturgill TW, Fisher TL, Blenis J and Lannigan DA (1998) pp90rsk1 regulates estrogen receptor-mediated transcription through phosphorylation of Ser-167. *Mol Cell Biol* **18**: 1978–1984
Jones RM, Branda J, Johnston KA, Polymenis M, Gadd M, Rustgi A, Callanan L and Schmidt EV (1996) An essential E box in the promoter of the gene encoding the mRNA cap-binding protein (eukaryotic initiation factor 4E) is a target for activation by c-myc. *Mol Cell Biol* **16**: 4754–4764
Karnik PS, Kulkarni S, Liu XP, Budd GT and Bukowski RM (1994) Estrogen receptor mutations in tamoxifen-resistant breast cancer. *Cancer Res* **54**: 349–353
Kato S, Endoh H, Masuhiro Y, Kitamoto T, Uchiyama S, Sasaki H, Masushige S, Gotoh Y, Nishida E and Kawashima H (1995) Activation of the estrogen receptor through phosphorylation by mitogen-activated protein kinase. *Science* **270**: 1491–1494
Kerekatte V, Smiley K, Hu B, Smith A, Gelder F and De Benedetti A (1995) The proto-oncogene/translation factor eIF4E: a survey of its expression in breast carcinomas. *Int J Cancer* **64**: 27–31
Klijn JG, Berns PM, Schmitz PI and Foekens JA (1992) The clinical significance of epidermal growth factor receptor (EGF-R) in human breast cancer: a review on 5232 patients. *Endocr Rev* **13**: 3–17
Knabbe C, Lippman ME, Wakefield LM, Flanders KC, Kasid A, Derynck R and Dickson RB (1987) Evidence that transforming growth factor-beta is a hormonally regulated negative growth factor in human breast cancer cells. *Cell* **48**: 417–428
Knabbe C, Kopp A, Hilgers W, Lang D, Muller V, Zugmaier G and Jonat W (1996) Regulation and role of TGF beta production in breast cancer. *Ann NY Acad Sci* **784**: 263–276
Knowlden JM, Gee JM, Seery LT, Farrow L, Gullick WJ, Ellis IO, Blamey RW, Robertson JF and Nicholson RI (1998) c-erbB3 and c-erbB4 expression is a feature of the endocrine responsive phenotype in clinical breast cancer. *Oncogene* **17**: 1949–1957
Kreipe H, Feist H, Fischer L, Felgner J, Heidorn K, Mettler L and Parwaresch R (1993) Amplification of c-myc but not of c-erbB-2 is associated with high proliferative capacity in breast cancer. *Cancer Res* **53**: 1956–1961
Kyprianou N, English HF, Davidson NE and Isaacs JT (1991) Programmed cell death during regression of the MCF-7 human breast cancer following estrogen ablation. *Cancer Res* **51**: 162–166
Lahooti H, Thorsen T and Aakvaag A (1998) Modulation of mouse estrogen receptor transcription activity by protein kinase C delta. *J Mol Endocrinol* **20**: 245–259
Lapidus RG, Ferguson AT, Ottaviano YL, Parl FF, Smith HS, Weitzman SA, Baylin SB, Issa JPJ and Davidson NE (1996) Methylation of estrogen and progesterone receptor gene 5′ CpG islands correlates with lack of estrogen and progesterone receptor gene expression in breast tumors. *Clin Cancer Res* **2**: 805–810
Lavinsky RM, Jepsen K, Heinzel T, Torchia J, Mullen TM, Schiff R, Del-Rio AL, Ricote M, Ngo S, Gemsch J, Hilsenbeck SG, Osborne CK, Glass CK, Rosenfeld MG and Rose DW (1998) Diverse signaling pathways modulate nuclear receptor recruitment of N-CoR and SMRT complexes. *Proc Natl Acad Sci USA* **95**: 2920–2925
Lavoie JN, Rivard N, L'Allemain G and Pouyssegur J (1996) A temporal and biochemical link between growth factor-activated MAP kinases, cyclin D1 induction and cell cycle entry. *Prog Cell Cycle Res* **2**: 49–58
Le Goff P, Montano MM, Schodin DJ and Katzenellenbogen BS (1994) Phosphorylation of the human estrogen receptor. Identification of hormone-regulated sites and examination of their influence on transcriptional activity. *J Biol Chem* **269**: 4458–4466
Lee AV, Weng CN, Jackson JG and Yee D (1997) Activation of estrogen receptor-mediated gene transcription by IGF-I in human breast cancer cells. *J Endocrinol* **152**: 39–47
Lehrer S, O'Shaughnessy J, Song HK, Levine E, Savoretti P, Dalton J, Lipsztein R,

 © 2000 Cancer Research Campaign

Kalnicki S and Bloomer WD (1989) Activity of pp60c-src protein kinase in human breast cancer. *Mt Sinai J Med* **56**: 83–85
Lewis TS, Shapiro PS and Ahn NG (1998) Signal tranduction through MAP kinase cascades. *Adv Cancer Res* **74**: 49–139
Leygue E, Dotzlaw H, Watson PH and Murphy LC (1998) Altered estrogen receptor alpha and beta messenger RNA expression during human breast tumorigenesis. *Cancer Res* **58**: 3197–3201
Li BD, Liu L, Dawson M and De Benedetti A (1997) Overexpression of eukaryotic initiation factor 4E (eIF4E) in breast carcinoma. *Cancer* **79**: 2385–2390
Li BD, McDonald JC, Nassar R and De Benedetti A (1998) Clinical outcome in stage I to III breast carcinoma and eIF4E overexpression. *Ann Surg* **227**: 756–761; discussion 761–763
Linardopoulos S, Malliri A, Pintzas A, Vassilaros S, Tsikkinis A and Spandidos DA (1990) Elevated expression of AP-1 activity in human breast tumors as compared to normal adjacent tissue. *Anticancer Res* **10**: 1711–1713
Locker AP, Birrell K, Bell JA, Nicholson RI, Elston CW, Blamey RW and Ellis IO (1992) Ki-67 immunoreactivity in breast carcinoma: relationships to prognostic variables and short term survival. *Eur J Surg Oncol* **18**: 224–229
Lu B, Leygue E, Dotzlaw H, Murphy LJ, Murphy LC and Watson PH (1998) Estrogen receptor-beta mRNA variants in human and murine tissues. *Mol Cell Endocrinol* **138**: 199–203
Lukas J, Bartkova J and Bartek J (1996) Convergence of mitogenic signalling cascades from diverse classes of receptors at the cyclin D-cyclin-dependent kinase-pRb-controlled G1 checkpoint. *Mol Cell Biol* **16**: 6917–6925
Lundy J, Schuss A, Stanick D, McCormack ES, Kramer S and Sorvillo JM (1991) Expression of neu protein, EGFR, and transforming growth factor alpha in breast cancer: correlation with clinicopathological parameters. *Am J Pathol* **138**: 1527–1534
Lupu R, Cardillo M, Harris L, Hijazi M and Rosenberg K (1995) Interaction between erbB-receptors and heregulin in breast cancer tumor progression and drug resistance. *Semin Cancer Biol* **6**: 135–145
Lupu R, Cardillo M, Cho C, Harris L, Hijazi M, Perez C, Rosenberg K, Yang D and Tang C (1996) The significance of heregulin in breast cancer tumor progression and drug resistance. *Breast Cancer Res Treat* **38**: 57–66
MacCallum J, Bartlett JM, Thompson AM, Keen JC, Dixon JM and Miller WR (1994) Expression of transforming growth factor beta mRNA isoforms in human breast cancer. *Br J Cancer* **69**: 1006–1009
MacCallum J, Keen JC, Bartlett JM, Thompson AM, Dixon JM and Miller WR (1996) Changes in expression of transforming growth factor beta mRNA isoforms in patients undergoing tamoxifen therapy. *Br J Cancer* **74**: 474–478
Maemura M, Akiyama SK, Woods VL Jr and Dickson RB (1995) Expression and ligand binding of alpha 2 beta 1 integrin on breast carcinoma cells. *Clin Exp Metastas* **13**: 223–235
Manning DL, McClelland RA, Gee JM, Chan CM, Green CD, Blamey RW and Nicholson RI (1993) The role of four oestrogen-responsive genes, pLIV1, pS2, pSYD3 and pSYD8, in predicting responsiveness to endocrine therapy in primary breast cancer. *Eur J Cancer* **29A**: 1462–1468
Manning DL, McClelland RA, Knowlden JM, Bryant S, Gee JMW, Gree CD, Robertson JFR, Blamey RW, Sutherland RL, Ormandy CJ and Nicholson RI (1995) Differential expression of oestrogen regulated genes in breast cancer. *Acta Oncol* **34**: 641–646
Marks JR, Huper G, Vaughn JP, Davis PL, Norris J, McDonnell DP, Wiseman RW, Futreal PA and Iglehart JD (1997) BRCA1 expression is not directly responsive to estrogen. *Oncogene* **14**: 115–121
Matsuda S, Kadowaki Y, Ichino M, Akiyama T, Toyoshima K and Yamamoto T (1993) 17 beta-estradiol mimics ligand activity of the c-erbB2 protooncogene product. *Proc Natl Acad Sci USA* **90**: 10803–10807
Matsui Y, Halter SA, Holt JT, Hogan BL and Coffey RJ (1990) Development of mammary hyperplasia and neoplasia in MMTV-TGF alpha transgenic mice. *Cell* **61**: 1147–1155
McClelland RA, Gee JMW, O'Sullivan L, Barnes DM, Robertson JFR, Ellis IO and Nicholson RI (1999) p21WAF1 expression and endocrine response in breast cancer. *J Pathol* (in press)
McDonnell DP, Vegeto E and O'Malley BW (1992) Identification of a negative regulatory function for steroid receptors. *Proc Natl Acad Sci USA* **89**: 10563–10567
McDonnell DP, Dana SL, Hoener PA, Lieberman BA, Imhof MO and Stein RB (1995) Cellular mechanisms which distinguish between hormone- and antihormone-activated estrogen receptor. *Ann N Y Acad Sci* **761**: 121–137
McInerney EM, Weis KE, Sun J, Mosselman S and Katzenellenbogen BS (1998) Transcription activation by the human estrogen receptor subtype beta (ER beta) studied with ER beta and ER alpha receptor chimeras. *Endocrinology* **139**: 4513–4522
Merkel DE and Osborne CK (1989) Prognostic factors in breast cancer. *Hematol Oncol Clin North Am* **3**: 641–652
Migliaccio A, Pagano M and Auricchio F (1993) Immediate and transient stimulation of protein tyrosine phosphorylation by estradiol in MCF-7 cells. *Oncogene* **8**: 2183–2191
Millon R, Nicora F, Muller D, Eber M, Klein-Soyer C and Abecassis J (1989) Modulation of human breast cancer cell adhesion by estrogens and antiestrogens. *Clin Exp Metastas* **7**: 405–415
Minden A, Lin A, Smeal T, Derijard B, Cobb M, Davis R and Karin M (1994) c-Jun N-terminal phosphorylation correlates with activation of the JNK subgroup but not the ERK subgroup of mitogen-activated protein kinases. *Mol Cell Biol* **14**: 6683–6688
Mohamood AS, Gyles P, Balan KV, Hollis VW, Eckberg WR, Asseffa A, Han Z, Wyche JH and Anderson WA (1997) Estrogen receptor, growth factor receptor and protooncogene protein activities and possible signal transduction crosstalk in estrogen dependent and independent breast cancer cell lines. *J Submicrosc Cytol Pathol* **29**: 1–17.
Morishita S, Niwa K, Ichigo S, Hori M, Murase T, Fujimoto J and Tamaya T (1995) Overexpressions of c-fos/jun mRNA and their oncoproteins (Fos/Jun) in the mouse uterus treated with three natural estrogens. *Cancer Lett* **97**: 225–231
Murphy LC, Dotzlaw H, Leygue E, Douglas D, Coutts A and Watson PH (1997) Estrogen receptor variants and mutations. *J Steroid Biochem Mol Biol* **62**: 363–372
Musgrove EA, Hamilton JA, Lee CS, Sweeney KJ, Watts CK and Sutherland RL (1993) Growth factor, steroid, and steroid antagonist regulation of cyclin gene expression associated with changes in T-47D human breast cancer cell cycle progression. *Mol Cell Biol* **13**: 3577–3587
Nakshatri H, Bhat-Nakshatri P, Martin DA, Goulet RJ Jr and Sledge GW Jr (1997) Constitutive activation of NF-kappaB during progression of breast cancer to hormone-independent growth. *Mol Cell Biol* **17**: 3629–3639
Nass SJ, Li M, Amundadottir LT, Furth PA and Dickson RB (1996) Role for Bcl-xL in the regulation of apoptosis by EGF and TGF beta 1 in c-myc overexpressing mammary epithelial cells. *Biochem Biophys Res Commun* **227**: 248–256
Nass SJ and Dickson RB (1998) Epidermal growth factor-dependent cell cycle progression is altered in mammary epithelial cells that overexpress c-myc. *Clin Cancer Res* **4**: 1813–1822
Nicholson RI, Wilson DW, Richards G, Griffiths K, Williams M, Elston CW and Blamey RW (1984) Biological and clinical aspects of oestrogen receptor measurements in rapidly progressing breast cancer. In: *Proc. IUPHAR 9th International Congress of Pharmacology*, Vol 3. Paton W, Mitchell J and Turner P (eds), pp. 75–79. McMillan Press: London
Nicholson RI, Colin P, Francis AB, Keshra R, Finlay P, Williams M, Elston CW, Blamey RW and Griffiths K (1986) Evaluation of an enzyme immunoassay for estrogen receptors in human breast cancers. *Cancer Res* **46**: 4299s–4302s
Nicholson RI, Eaton CL and Manning DL (199?) New developments in the endocrine management of breast cancer. In: *Recent Advances in Endocrinology and Metabolism*, Vol 4, Edwards CRW and Lincoln DW (eds), pp. 151–165. Churchill Livingstone: Edinburgh
Nicholson RI, Bouzubar N, Walker KJ, McClelland R, Dixon AR, Robertson JF, Ellis IO and Blamey RW (1991) Hormone sensitivity in breast cancer: influence of heterogeneity of oestrogen receptor expression and cell proliferation. *Eur J Cancer* **27**: 908–913
Nicholson RI, McClelland RA, Finlay P, Eaton CL, Gullick WJ, Dixon AR, Robertson JFR, Ellis IO and Blamey RW (1993) Relationship between EGF-R, c-erbB-2 protein expression and Ki67 immunostaining in breast cancer and hormone sensitivity. *Eur J Cancer* **29A**: 1018–1023
Nicholson RI, McClelland RA, Gee JM, Manning DL, Cannon P, Robertson JF, Ellis IO and Blamey RW (1994) Transforming growth factor-alpha and endocrine sensitivity in breast cancer. *Cancer Res* **54**: 1684–1689
Nicholson RI, McClelland RA, Gee JMW, Manning DL, Cannon P, Robertson JFR, Ellis IO and Blamey RW (1994) Epidermal growth factor receptor expression in breast cancer: association with response to endocrine therapy. *Breast Cancer Res Treat* **29**: 117–125
Nicholson RI, McClelland RA and Gee JM (1995) Steroid hormone receptors and their clinical significance in cancer. *J Clin Pathol* **48**: 890–895
Nicholson RI and Gee JWM (1996) Growth factors and modulation of endocrine response in breast cancer. In: *Hormones and Cancer*, Vedeckis WV (ed), pp. 227–261. Birkhauser: Boston
Nicholson RI, Gee JMW, Harper ME, Ellis IO, Willsher P and Robertson JFR (1997*a*) erbB signalling in clinical breast cancer: relationship to endocrine sensitivity. *Endocrine-Related Cancer* **4**: 297–306
Nicholson RI, Gee JMW, Jones H, Harper ME, Wakeling AE, Willsher P and Robertson JFR (1997*b*) erbB signalling and endocrine sensitivity of human breast cancer. In: *EGF Receptor in Tumour Growth and Progression*, Harkin et al (eds) pp. 105–128 Springer Verlag: Bosten

*© 2000 Cancer Research Campaign* 

Nicholson RI, Gee JMW, Seery LT, McClelland RA, Harper ME, Holt B, Barnes D, Robertson JFR, Pinder S and Ellis IO (1997*c*) p53 protein expression in human breast cancer: Relationship to tumour differentiation and endocrine response. ESO – Special Scientific Reports

Nicholson RI, Robertson JFR, Seery LT and Gee JMW (1999*a*) Endocrine response and failure in breast cancer: a role for the interplay of steroid and growth factor signalling pathways and therapeutic implications. In: Furr BJA and Jordan, VC (eds) *Pharmacological Handbook* (in press)

Nicholson RI, McClelland RA, Robertson JFR and Gee JMW (1999*b*) Involvement of steroid hormone and growth factor cross-talk in endocrine response in breast cancer. *Endocrine-Related Cancer* (in press)

Nicholson S, Halcrow P, Fardon JR, Sainsbury JRC, Chambers P and Harris AL (1989) Expression of epidermal growth factor receptors associated with lack of response to endocrine therapy in recurrent breast cancer. *Lancet **I*** 182–185

Osin P, Gusterson BA, Philp E, Waller J, Bartek J, Peto J and Crook T (1998) Predicted anti-oestrogen resistance in BRCA-associated familial breast cancers. *Eur J Cancer* **34**: 1683–1686

Ottaviano YL, Issa JP, Parl FF, Smith HS, Baylin SB and Davidson NE (1994) Methylation of the estrogen receptor gene CpG island marks loss of estrogen receptor expression in human breast cancer cells. *Cancer Res* **54**: 2552–2555

Paech K, Webb P, Kuiper GG, Nilsson S, Gustafsson J, Kushner PJ and Scanlan TS (1997) Differential ligand activation of estrogen receptors ERalpha and ERbeta at AP1 sites. *Science* **277**: 1508–1510

Papa V, Gliozzo B, Clark GM, McGuire WL, Moore D, Fujita-Yamaguchi Y, Vigneri R, Goldfine ID and Pezzino V (1993) Insulin-like growth factor-I receptors are overexpressed and predict a low risk in human breast cancer. *Cancer Res* **53**: 3736–3740

Parker MG (1998) Transcriptional activation by oestrogen receptors. *Biochem Soc Symp* **63**: 45–50

Paul A, Wilson S, Belham CM, Robinson CJ, Scott PH, Gould GW and Plevin R (1997) Stress-activated protein kinases: activation, regulation and function. *Cell Signal* **9**: 403–410

Perry RR, Kang Y and Greaves BR (1995) Relationship between tamoxifen-induced transforming growth factor beta 1 expression, cytostasis and apoptosis in human breast cancer cells. *Br J Cancer* **72**: 1441–1446

Pfahl M (1993) Nuclear receptor/AP-1 interaction. *Endocr Rev* **14**: 651–658. of c-Myc. *FEBS Lett* **353(3)**: 281–5

Philips A, Chalbos D and Rochefort H (1993) Estradiol increases and anti-estrogens antagonize the growth factor-induced activator protein-1 activity in MCF7 breast cancer cells without affecting c-fos and c-jun synthesis. *J Biol Chem* **268**: 14103–14108

Pietras RJ, Arboleda J, Reese DM, Wongvipat N, Pegram MD, Ramos L, Gorman CM, Parker MG, Sliwkowski MX and Slamon DJ (1995) HER-2 tyrosine kinase pathway targets estrogen receptor and promotes hormone-independent growth in human breast cancer cells. *Oncogene* **10**: 2435–2446

Porter W, Saville B, Hoivik D and Safe S (1997) Functional synergy between the transcription factor Sp1 and the estrogen receptor. *Mol Endocrinol* **11**: 1569–1580

Prall OW, Rogan EM and Sutherland RL (1998a) Estrogen regulation of cell cycle progression in breast cancer cells. *J Steroid Biochem Mol Biol* **65**: 169–174

Prall OW, Rogan EM, Musgrove EA, Watts CK and Sutherland RL (1998b) c-Myc or cyclin D1 mimics estrogen effects on cyclin E-Cdk2 activation and cell cycle reentry. *Mol Cell Biol* **18**: 4499–4508

Ram TG, Kokeny KE, Dilts CA and Ethier SP (1995) Mitogenic activity of neu differentiation factor/heregulin mimics that of epidermal growth factor and insulin-like growth factor-I in human mammary epithelial cells. *J Cell Physiol* **163**: 589–596

Ray P, Ghosh SK, Zhang DH and Ray A (1997) Repression of interleukin-6 gene expression by 17 beta-estradiol: inhibition of the DNA-binding activity of the transcription factors NF-IL6 and NF-kappa B by the estrogen receptor. *FEBS Lett* **409**: 79–85

Reiss K, Ferber A, Travali S, Porcu P, Phillips PD, Baserga R (1991) The protooncogene c-myb increases the expression of insulin-like growth factor 1 and insulin-like growth factor 1 receptor messenger RNAs by a transcriptional mechanism. *Cancer Res* **51**: 5997–6000

Reiss M and Barcellos-Hoff MH (1997) Transforming growth factor-beta in breast cancer: a working hypothesis. *Breast Cancer Res Treat* **45**: 81–95

Richards RG, DiAugustine RP, Petrusz P, Clark GC and Sebastian J (1996) Estradiol stimulates tyrosine phosphorylation of the insulin-like growth factor-1 receptor and insulin receptor substrate-1 in the uterus. *Proc Natl Acad Sci USA* **93**: 12002–12007

Robertson JF (1996) Oestrogen receptor: a stable phenotype in breast cancer. *Br J Cancer* **73**: 5–12

Robertson JF, Williams MR, Todd J, Nicholson RI, Morgan DA and Blamey RW (1989) Factors predicting the response of patients with advanced breast cancer to endocrine (Megace) therapy. *Eur J Cancer Clin Oncol* **25**: 469–475

Robertson JF, Bates K, Pearson D, Blamey RW and Nicholson RI (1992) Comparison of two oestrogen receptor assays in the prediction of the clinical course of patients with advanced breast cancer. *Br J Cancer* **65**: 727–730

Rochefort H (1995) Oestrogen- and anti-oestrogen-regulated genes in human breast cancer. *Ciba Found Symp* **191**: 254–265

Ross JS and Fletcher JA (1998) The HER-2/neu oncogene in breast cancer: prognostic factor, predictive factor, and target for therapy. *Stem Cells* **16**: 413–428

Rubino D, Driggers P, Arbit D, Kemp L, Miller B, Coso O, Pagliai K, Gray K, Gutkind S and Segars J (1998) Characterization of Brx, a novel Dbl family member that modulates estrogen receptor action. *Oncogene* **16**: 2513–2526

Sala A and Calabretta B (1992) Regulation of BALB/c 3T3 fibroblast proliferation by B-myb is accompanied by selective activation of cdc2 and cyclin D1 expression. *Proc Natl Acad Sci USA* **89**: 10415–10419

Salomon DS, Kim N, Saeki T and Ciardiello F (1990) Transforming growth factor-alpha: an oncodevelopmental growth factor. *Cancer Cells* **2**: 389–397

Sandgren EP, Luetteke NC, Palmiter RD, Brinster RL and Lee DC (1990) Overexpression of TGF alpha in transgenic mice: induction of epithelial hyperplasia, pancreatic metaplasia, and carcinoma of the breast. *Cell* **61**: 1121–1135

Santoni-Rugiu E, Jensen MR and Thorgeirsson SS (1998) Disruption of the pRb/E2F pathway and inhibition of apoptosis are major oncogenic events in liver constitutively expressing c-myc and transforming growth factor alpha. *Cancer Res* **58**: 123–134

Seery LT, Gee JMW, Dewhurst OL and Nicholson RI. Molecular mechanisms of antioestrogen action. In: *Handbook of Experimental Pharmacology*, Vol 35 (in press)

Seery LT, Knowlden JM, Gee JMW, Robertson JFR, Kenny FS, Ellis IO and Nicholson RI (1999) BRAC1 expression levels predict distant metastasis of sporadic breast cancer. *Int J Cancer* (in press)

Seshadri R, Firgaira FA, Horsfall DJ, McCaul K, Setlur V and Kitchen P (1993) Clinical significance of HER-2/neu oncogene amplification in primary breast cancer. The South Australian Breast Cancer Study Group. *J Clin Oncol* **11**: 1936–1942

Sharma AK, Horgan K, Douglas-Jones A, McClelland R, Gee J and Nicholson RI (1994a) Dual immunocytochemical analysis of oestrogen and epidermal growth factor receptors in human breast cancer. *Br J Cancer* **69**: 1032–1037

Sharma AK, Horgan K, McClelland RA, Douglas-Jones AG, Van Agthoven T, Dorssers LC and Nicholson RI (1994*b*) A dual immunocytochemical assay for oestrogen and epidermal growth factor receptors in tumour cell lines. *Histochem J* **26**: 306–310

Sharma H and Narayanan R (1996) The NF-kappaB transcription factor in oncogenesis. *Anticancer Res* **16**: 589–596

Sivaraman VS, Wang H, Nuovo GJ and Malbon CC (1997) Hyperexpression of mitogen-activated protein kinase in human breast cancer. *J Clin Invest* **99**: 1478–1483

Smith CL, Conneely OM and O'Malley BW (1993) Modulation of the ligand-independent activation of the human estrogen receptor by hormone and antihormone. *Proc Natl Acad Sci USA* **90**: 6120–6124

Smith CL, Onate SA, Tsai MJ and O'Malley BW (1996) CREB binding protein acts synergistically with steroid receptor coactivator-1 to enhance steroid receptor-dependent transcription. *Proc Natl Acad Sci USA* **93**: 8884–8888

Smith CL, Nawaz Z and O'Malley BW (1997) Coactivator and corepressor regulation of the agonist/antagonist activity of the mixed antiestrogen, 4-hydroxytamoxifen. *Mol Endocrinol* **11**: 657–666

Sonenberg N and Gingras AC (1998) The mRNA 5′ cap-binding protein eIF4E and control of cell growth. *Curr Opin Cell Biol* **10**: 268–275

Sorrells DL, Black DR, Meschonat C, Rhoads R, De Benedetti A, Gao M, Williams BJ and Li BD (1998) Detection of eIF4E gene amplification in breast cancer by competitive PCR. *Ann Surg Oncol* **5**: 232–237

Sovak MA, Bellas RE, Kim DW, Zanieski GJ, Rogers AE, Traish AM and Sonenshein GE (1997) Aberrant nuclear factor-kappaB/Rel expression and the pathogenesis of breast cancer. *J Clin Invest* **100**: 2952–2960

Spillman MA and Bowcock AM (1996) BRCA1 and BRCA2 mRNA levels are coordinately elevated in human breast cancer cells in response to estrogen. *Oncogene* **13**: 1639–1645

Suen CS, Berrodin TJ, Mastroeni R, Cheskis BJ, Lyttle CR and Frail DE (1998) A transcriptional coactivator, steroid receptor coactivator-3, selectively augments steroid receptor transcriptional activity. *J Biol Chem* **273**: 27645–27653

Sun G, Porter W and Safe S (1998) Estrogen-induced retinoic acid receptor alpha 1 gene expression: role of estrogen receptor-Sp1 complex. *Mol Endocrinol* **12**: 882–890

 © 2000 Cancer Research Campaign

Sutherland RL, Hamilton JA, Sweeney KJ, Watts CK and Musgrove EA (1995) Expression and regulation of cyclin genes in breast cancer. *Acta Oncol* **34**: 651–656

Thompson AM, Kerr DJ and Steel CM (1991) Transforming growth factor beta 1 is implicated in the failure of tamoxifen therapy in human breast cancer. *Br J Cancer* **63**: 609–614

Thompson MA, Rosenthal MA, Ellis SL, Friend AJ, Zorbas MI, Whitehead RH and Ramsay RG (1998) c-Myb down-regulation is associated with human colon cell differentiation, apoptosis, and decreased Bcl-2 expression. *Cancer Res* **58**: 5168–5175

Tiniakos DG, Scott LE, Corbett IP, Piggott NH and Horne CH (1994) Studies of c-jun oncogene expression in human breast using a new monoclonal antibody, NCL-DK4. *J Pathol* **172**: 19–26

Travers MT, Barrett-Lee PJ, Berger U, Luqmani YA, Gazet JC, Powles TJ and Coombes RC (1988) Growth factor expression in normal, benign, and malignant breast tissue. *Br Med J (Clin Res Ed)* **296**: 1621–1624

Troppmair J, Bruder JT, Munoz H, Lloyd PA, Kyriakis J, Banerjee P, Avruch J and Rapp UR (1994) Mitogen-activated protein kinase/extracellular signal-regulated protein kinase activation by oncogenes, serum, and 12-*O*-tetradecanoylphorbol-13-acetate requires Raf and is necessary for transformation. *J Biol Chem* **269**: 7030–7035

Trowbridge JM, Rogatsky I and Garabedian MJ (1997) Regulation of estrogen receptor transcriptional enhancement by the cyclin A/Cdk2 complex. *Proc Natl Acad Sci USA* **94**: 10132–10137

Tzahar E, Waterman H, Chen X, Levkowitz G, Karunagaran D, Lavi S, Ratzkin BJ and Yarden Y (1996) A hierarchical network of interreceptor interactions determines signal transduction by Neu differentiation factor/neuregulin and epidermal growth factor. *Mol Cell Biol* **16**: 5276–5287

Tzahar E and Yarden Y (1998) The ErbB-2/HER2 oncogenic receptor of adenocarcinomas: from orphanhood to multiple stromal ligands. *Biochim Biophys Acta* **1377**: M25–M37

Tzukerman MT, Esty A, Santiso-Mere D, Danielian P, Parker MG, Stein RB, Pike JW and McDonnell DP (1994) Human estrogen receptor transactivational capacity is determined by both cellular and promoter context and mediated by two functionally distinct intramolecular regions. *Mol Endocrinol* **8**: 21–30

Umekita Y, Enokizono N, Sagara Y, Kuriwaki K, Takasaki T, Yoshida A and Yoshida H (1992) Immunohistochemical studies on oncogene products (EGF-R, c-erbB-2) and growth factors (EGF, TGF-alpha) in human breast cancer: their relationship to oestrogen receptor status, histological grade mitotic index and nodal status. *Virchows Arch A Pathol Anat Histopathol* **420**: 345–351

Van Roy F, Mareel M, Vleminckx K, Beyaert R, Fiers W, Devleeschouwer N, Muquardt C, Legros N, Bracke M and Leclercq G (1990) Hormone sensitivity in vitro and in vivo of v-ras-transfected MCF-7 cell derivatives. *Int J Cancer* **46**: 522–532

Vladusic EA, Hornby AE, Guerra-Vladusic FK and Lupu R (1998) Expression of estrogen receptor beta messenger RNA variant in breast cancer. *Cancer Res* **58**: 210–214

Walker KJ, Price-Thomas JM, Candlish W and Nicholson RI (1991) Influence of the antioestrogen tamoxifen on normal breast tissue. *Br J Cancer* **64**: 764–768

Walker KJ, McClelland RA, Candlish W, Blamey RW and Nicholson RI (1992) Heterogeneity of oestrogen receptor expression in normal and malignant breast tissue. *Eur J Cancer* **28**: 34–37

Walker RA and Dearing SJ (1992) Transforming growth factor beta 1 in ductal carcinoma in situ and invasive carcinomas of the breast. *Eur J Cancer* **28**: 641–644

Walker RA, Jones JL, Chappell S, Walsh T and Shaw JA (1997) Molecular pathology of breast cancer and its application to clinical management. *Cancer Metastas Rev* **16**: 5–27

Wallasch C, Weiss FU, Niederfellner G, Jallal B, Issing W and Ullrich A (1995) Heregulin-dependent regulation of HER2/neu oncogenic signaling by heterodimerization with HER3. *EMBO J* **14**: 4267–4275

Wang C, Thor AD, Moore DH 2nd, Zhao Y, Kerschmann R, Stern R, Watson PH and Turley EA (1998) The overexpression of RHAMM, a hyaluronan-binding protein that regulates ras signaling, correlates with overexpression of mitogen-activated protein kinase and is a significant parameter in breast cancer progression. *Clin Cancer Res* **4**: 567–576

Wang Q, Maloof P, Wang H, Fenig E, Stein D, Nichols G, Denny TN, Yahalom J and Wieder R (1998) Basic fibroblast growth factor downregulates Bcl-2 and promotes apoptosis in MCF-7 human breast cancer cells. *Exp Cell Res* **238**: 177–187

Wasylyk B, Hagman J and Gutierrez-Hartmann A (1998) Ets transcription factors: nuclear effectors of the Ras-MAP-kinase signaling pathway. *Trends Biochem Sci* **23**: 213–216

Watanabe T, Inoue S, Ogawa S, Ishii Y, Hiroi H, Ikeda K, Orimo A and Muramatsu M (1997) Agonistic effect of tamoxifen is dependent on cell type, ERE-promoter context, and estrogen receptor subtype: functional difference between estrogen receptors alpha and beta. *Biochem Biophys Res Commun* **236**: 140–145

Watson DM, Elton RA, Jack WJ, Dixon JM, Chetty U and Miller WR (1991) The H-ras oncogene product p21 and prognosis in human breast cancer. *Breast Cancer Res Treat* **17**: 161–169

Webb P, Lopez GN, Uht RM and Kushner PJ (1995) Tamoxifen activation of the estrogen receptor/AP-1 pathway: potential origin for the cell-specific estrogen-like effects of antiestrogens. *Mol Endocrinol* **9**: 443–456

Weisberg E, Sattler M, Ewaniuk DS and Salgia R (1997) Role of focal adhesion proteins in signal transduction and oncogenesis. *Crit Rev Oncog* **8**: 343–358

Werner H and Le Roith D (1997) The insulin-like growth factor-I receptor signaling pathways are important for tumorigenesis and inhibition of apoptosis. *Crit Rev Oncog* **8**: 71–92

Westley BR, Clayton SJ, Daws MR, Molloy CA and May FE (1998) Interactions between the oestrogen and insulin-like growth factor signalling pathways in the control of breast epithelial cell proliferation. *Biochem Soc Symp* **63**: 35–44

Whitmarsh AJ and Davis RJ (1996) Transcription factor AP-1 regulation by mitogen-activated protein kinase signal transduction pathways. *J Mol Med* **74**: 589–607

Wilcken NRC, Prall OWJ, Musgrove EA and Sutherland RL (1997) Inducible overexpression of cyclin D1 in breast cancer cells reverses the growth-inhibitory effects of antiestrogens. *Clin Cancer Res* **3**: 849–854

Williams MR, Todd JH, Nicholson RI, Elston CW, Blamey RW and Griffiths K (1986) Survival patterns in hormone treated advanced breast cancer. *Br J Surg* **73**: 752–755

Wiseman LR, Johnson MD, Wakeling AE, Lykkesfeldt AE, May FE and Westley BR (1993) Type IIGF receptor and acquired tamoxifen resistance in oestrogen-responsive human breast cancer cells. *Eur J Cancer* **29A**: 2256–2264

Wosikowski K, Eppenberger U, Kung W, Nagamine Y and Mueller H (1992) c-fos, c-jun and c-myc expressions are not growth rate limiting for the human MCF-7 breast cancer cells. *Biochem Biophys Res Commun* **188**: 1067–1076

Xie W, Duan R and Safe S (1999) Estrogen induces adenosine deaminase gene expression in MCF-7 human breast cancer cells: role of estrogen receptor-Sp1 interactions. *Endocrinology* **140**: 219–227

Yee D (1998) The insulin-like growth factors and breast cancer – revisited. *Breast Cancer Res Treat* **47**: 197–199

Zwijsen RM, Wientjens E, Klompmaker R, van der Sman J, Bernards R and Michalides RJ (1997) CDK-independent activation of estrogen receptor by cyclin D1. *Cell* **88**: 405–415

Zwijsen RM, Buckle RS, Hijmans EM, Loomans CJ and Bernards R (1998) Ligand-independent recruitment of steroid receptor coactivators to estrogen receptor by cyclin D1. *Genes Dev* **12**: 3488–3498

*© 2000 Cancer Research Campaign*
